# Oligodendroglial fatty acid metabolism as a central nervous system energy reserve

**DOI:** 10.1038/s41593-024-01749-6

**Published:** 2024-09-09

**Authors:** Ebrahim Asadollahi, Andrea Trevisiol, Aiman S. Saab, Zoe J. Looser, Payam Dibaj, Reyhane Ebrahimi, Kathrin Kusch, Torben Ruhwedel, Wiebke Möbius, Olaf Jahn, Jun Yup Lee, Anthony S. Don, Michelle-Amirah Khalil, Karsten Hiller, Myriam Baes, Bruno Weber, E. Dale Abel, Andrea Ballabio, Brian Popko, Celia M. Kassmann, Hannelore Ehrenreich, Johannes Hirrlinger, Klaus-Armin Nave

**Affiliations:** 1 Max Planck Institute for Multidisciplinary Sciences, Department of Neurogenetics, Göttingen, Germany; 2 University of Toronto, Sunnybrook Health Sciences Centre, Department of Physical Sciences, North York, Ontario Canada; 3 University of Zurich, Institute of Pharmacology and Toxicology, Zurich, Switzerland; 4 Center for Rare Diseases Göttingen, Department of Pediatrics and Pediatric Neurology, Georg August University Göttingen, Göttingen, Germany; 5 University of Göttingen Medical School, Institute for Auditory Neuroscience and Inner Ear Lab, Göttingen, Germany; 6 Max Planck Institute for Multidisciplinary Sciences, Department of Molecular Neurobiology, Neuroproteomics Group, Göttingen, Germany; 7 University Medical Center Göttingen, Department of Psychiatry and Psychotherapy, Translational Neuroproteomics Group, Göttingen, Germany; 8 School of Medical Sciences and Charles Perkins Centre, The University of Sydney, Camperdown, New South Wales Australia; 9 Department for Bioinformatics and Biochemistry, Braunschweig Integrated Center of System Biology, Technische Universität Braunschweig, Braunschweig, Germany; 10 Lab of Cell Metabolism, Department of Pharmaceutical and Pharmacological Sciences, KU Leuven, Leuven, Belgium; 11 Department of Medicine, David Geffen School of Medicine at UCLA, Los Angeles, CA USA; 12 Telethon Institute of Genetics and Medicine, Naples, Italy; 13 Department of Translational Medical Sciences, Federico II University, Naples, Italy; 14 Department of Molecular and Human Genetics, Baylor College of Medicine, Houston, TX USA; 15 Jan and Dan Duncan Neurological Research Institute, Texas Children’s Hospital, Houston, TX USA; 16 Feinberg School of Medicine, Northwestern University, Chicago, IL USA; 17 Max Planck Institute for Multidisciplinary Sciences, Clinical Neuroscience, Göttingen, Germany; 18 Central Institute of Mental Health, Mannheim, Germany; 19 Carl-Ludwig-Institute for Physiology, Faculty of Medicine, University of Leipzig, Leipzig, Germany

**Keywords:** Oligodendrocyte, Cellular neuroscience

## Abstract

Brain function requires a constant supply of glucose. However, the brain has no known energy stores, except for glycogen granules in astrocytes. In the present study, we report that continuous oligodendroglial lipid metabolism provides an energy reserve in white matter tracts. In the isolated optic nerve from young adult mice of both sexes, oligodendrocytes survive glucose deprivation better than astrocytes. Under low glucose, both axonal ATP levels and action potentials become dependent on fatty acid β-oxidation. Importantly, ongoing oligodendroglial lipid degradation feeds rapidly into white matter energy metabolism. Although not supporting high-frequency spiking, fatty acid β-oxidation in mitochondria and oligodendroglial peroxisomes protects axons from conduction blocks when glucose is limiting. Disruption of the glucose transporter GLUT1 expression in oligodendrocytes of adult mice perturbs myelin homeostasis in vivo and causes gradual demyelination without behavioral signs. This further suggests that the imbalance of myelin synthesis and degradation can underlie myelin thinning in aging and disease.

## Abstract (web summary)

Brain functions require a constant supply of glucose. However, the brain energy stores are unclear. Here, the authors show that oligodendroglial fatty acid metabolism can be an energy reserve for white matter axons, supporting their function.

## Main

In the central nervous system of vertebrates, oligodendrocytes make myelin to enable saltatory impulse conduction^1^. Myelinating oligodendrocytes also provide fast spiking axons with lactate or pyruvate^2–4^ for the generation of ATP^5^. This metabolic support of axonal projections by the associated glial cells has preceded the evolution of myelin in vertebrates^6,7^, but is most important when myelin deprives axons from access to metabolites of the extracellular milieu. In nonmyelinating species, axon-associated glial cells also harbor lipid droplets^8^, which can serve as local energy reserves by mobilizing fatty acids (FAs) under starvation conditions^9^.

Vertebrate myelin is a multilayered, highly lipid-rich membrane compartment^10^ that is more dynamic than previously thought. Maintaining myelin maintenance throughout adult life requires its constant turnover, including high-level expression of myelin proteins and their incorporation into the myelin sheath^11,12^. Similarly, myelin lipids are subject to rapid turnover, which has been difficult to quantify by metabolic labeling studies^13^, because FAs and their breakdown products are efficiently reutilized.

In oligodendrocytes, FA β-oxidation takes place in mitochondria and peroxisomes, the latter being prevalent within the myelin compartment^14^. Mitochondrial acetyl-CoA can be either metabolized for the generation of ATP (oxidative phosphorylation (OXPHOS)) or released to the cytoplasm via the citric acid shuttle^15^ and locally recycled in FA synthesis. Mitochondrial acetyl-CoA can also be used for ketogenesis^16^.

**Fig. 1:**
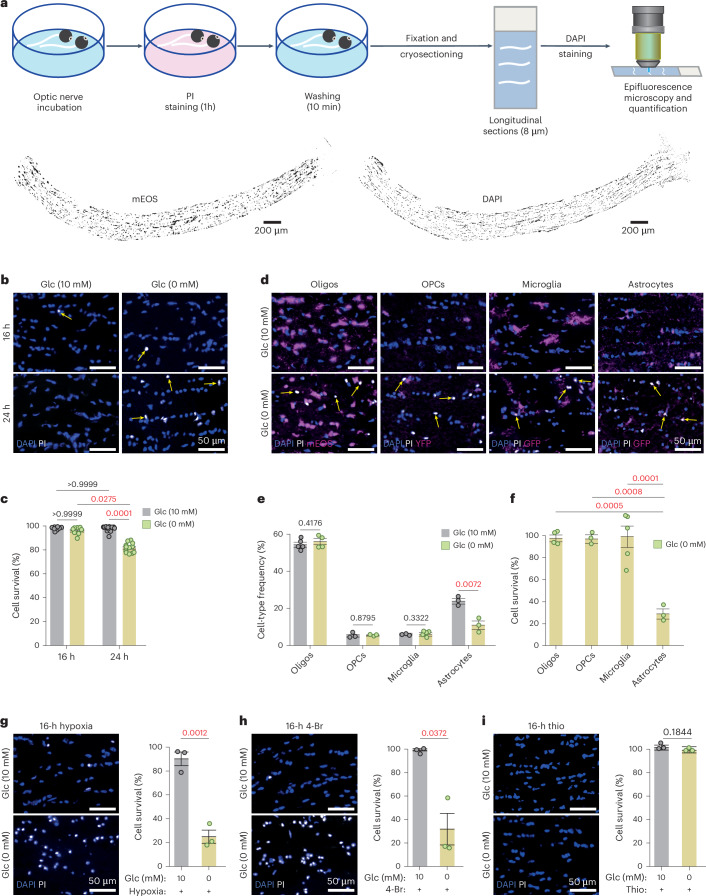
Optic nerve glial cell survival under glucose deprivation requires FA utilization. **a**, Schematic representation of the experimental pipeline. Below, myelinated optic nerves from *Cnp-mEOS* reporter mice, maintained ex vivo (*n* = 5). Left, longitudinal section showing mEOS^+^ oligodendrocytes (black on white). Right, all DAPI^+^ cell nuclei (black on white). **b**, Higher magnification of DAPI^+^ (blue) optic nerve glia and PI^+^ (white) dying cells (arrows). Left, cells surviving in 10 mM glucose (Glc). Right, without glucose, cells surviving up to 16 h, but many dying after 24 h (quantified in **c**). **c**, Cell survival quantified by subtracting (PI^+^DAPI^+^) dying cells from total (DAPI^+^) cells including data from **d**–**f** (16 h: *N* = *n* = 8; 24 h: *N* = *n* = 12; mean ± s.e.m., Kruskal–Wallis test, Dunn’s multiple comparison). **d**, Different vulnerabilities of glial subtypes to 24-h glucose withdrawal. Optic nerve longitudinal sections are labeled by DAPI (all cells), PI (dying cells) and genetically expressed cell-specific markers (oligodendrocytes: *Cnp*-PTS1-mEOS; OPCs: *Ng2*-YFP; microglia: *Cxcr1*-GFP; astrocytes: *Aldh1l1*-GFP). Note the shrunken cell nuclei in glucose-free medium and lack of overlap between oligodendrocytes and dying cells. **e**, Frequency of glial subtypes after 24-h incubation in 10 mM or 0 mM glucose (oligodendrocytes: 10 mM, *N* = *n* = 5; 0 mM, *N* = *n* = 4; microglia: 10 mM, *N* = *n* = 3; 0 mM, *N* = *n* = 5; OPCs and astrocytes: *N* = *n* = 3, both) with data from **d**. **f**, Survival rate of glial subtypes after 24 h in 10 mM or 0 mM glucose, normalized to cells in glucose-containing aCSF (100%) with data from **e** (mean ± s.e.m., one-way ANOVA, Tukey’s multiple comparison). **g**, Glial cells that survive 16-h glucose deprivation dying under hypoxia (bottom), demonstrating oxidation of an endogenous energy reserve other than glucose (*N* = *n* = 3, each). **h**, 4-Br (a mitochondrial β-oxidation inhibitor) treatment of glucose-deprived optic nerves causing widespread cell death, demonstrating FAs as an energy reserve (*N* = *n* = 3). Note that 4-Br is not cytotoxic by itself (mean ± s.e.m., unpaired, two-tailed Student’s *t*-test, heteroscedastic). **i**, Thio (a peroxisomal β-oxidation inhibitor) treatment not increasing glial death which indicates mitochondrial β-oxidation sufficient for glial survival (*N* = *n* = 3). Animals, both sexes, are aged 2 months. Percentages (in **g**–**i**) were calculated relative to overall survival for 16 h with glucose under normoxia (in **c**). *N*, individual optic nerves; *n*, independent experiments. Error bars in **e**, **g** and **i**: mean ± s.e.m., unpaired, two-tailed Student’s *t*-test. Source data

Conceivably, reduced glucose availability lowers acetyl-CoA and FA synthesis and should affect lipid metabolism and myelin turnover. In a range of neurodegenerative disorders, including Alzheimer’s disease, the reduction of brain glucose metabolism correlates with white matter abnormalities^17^. To address the question of whether myelin lipid synthesis, FA turnover and oligodendroglial energy metabolism are indeed interconnected, we chose the acutely isolated optic nerve from young adult mice (Fig. 1a) as a model system. We assessed axonal conductivity and ATP levels in myelinated optic nerves under defined metabolic conditions, including low glucose, and in the presence of specific metabolic inhibitors, complemented by oligodendrocyte-specific, gene-targeting experiments in vivo. The latter allowed us to detect a gradual loss of myelin membranes when oligodendrocytes have reduced glucose availability. More importantly, in the present study we show that oligodendroglial FA metabolism can be an energy reserve for white matter axons, supporting their function.

## Results

### Glial cells in glucose-deprived optic nerves rely on FA β-oxidation

We analyzed fully myelinated transgenic mice (both sexes) at age 2 months, expressing fluorescent proteins in mature oligodendrocytes (*Cnp-mEos2-PTS1*)^14^ or astrocytes (*Aldh1L1-GFP*). Optic nerves were incubated at 37 °C in artificial cerebrospinal fluid (aCSF) containing 10 mM glucose, 0 mM glucose (termed ‘glucose free’) or low glucose (termed ‘starved’; Methods). After 24 h, the total number of cells (DAPI^+^), the number of dying cells (propidium iodide positive (PI^+^)) and the identity of surviving cells were determined by fluorescence analysis of sectioned nerves. Surprisingly, the large majority (>97%) of oligodendrocytes appeared healthy after 24 h in glucose-free medium, whereas >70% of astrocytes had died (Fig. 1b-f). Oligodendrocyte precursor cells (OPCs) and microglia were also not reduced. Next, we compared earlier time points and found no cell death at 16 h (Fig. 1c). This suggests that all glial cells in the myelinated optic nerve can principally survive in the absence of glucose by utilizing a pre-existing energy reserve.

**Extended Data Fig. 1:**
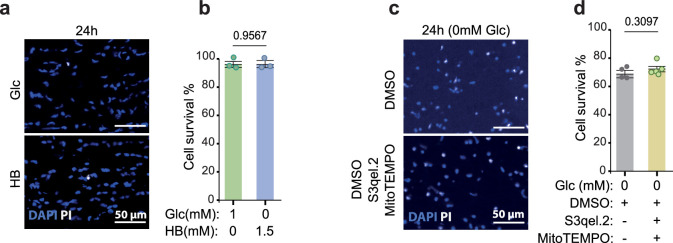
Death of glucose-deprived optic nerve glial cells is not caused by oxidative stress. **a**, Acutely isolated mouse optic nerves (age 2 months) were maintained for 24 h either in the presence of low (1 mM) glucose or 1.5 mM beta-hydroxybutyrate (HB). Longitudinal nerve sections were stained with DAPI (blue) and propidium iodide (in white). **b**, Bar graphs showing the percentage of viable cells for each condition (compare to Fig. 1c). Normal survival is possible in the presence of 1 mM glucose or 1.5 mM HB, indicating the critical role of glucose in energy production (N = n = 3 for both conditions; mean ± SEM, unpaired two-sided t-test). **c**, Longitudinal sections of optic nerves stained with PI and DAPI after 24 h incubation in glucose-free aCSF and in the presence of 10 μM of S3qel-2 (inhibitor for ROS production in complex III of electron transport chain) and 10 μM MitoTEMPO (mitochondrial ROS scavenger) or vehicle (DMSO) as control. **d**, Quantified cell survival (PI/DAPI) indicates that ROS do not contribute to cell death (N = n = 4 for control and N = n = 5 for ROS inhibitor/scavenger; wild type mice, 8-10 weeks old; mean ± SEM, unpaired two-sided t-test). N and n indicate the total number of optic nerves used for each condition and the total number of independent experiments, respectively. Source data

In the presence of 1 mM glucose, a concentration insufficient to maintain axonal conduction (see also below for nerve function), all cells of the optic nerve stayed alive for at least 24 h. Glucose is essential for the pentose phosphate pathway and the synthesis of nucleotides. To rule out glucose-free medium being detrimental independent of the reduced energy metabolism, we incubated optic nerves in aCSF containing as little as 1.5 mM 3-hydroxybutyrate as an alternative energy source and detected no cell death after 24 h (Extended Data Fig. 1a,b). Moreover, optic nerves that were kept glucose free in the presence of a reactive oxygen species (ROS) inhibitor (S3QEL-2) and a mitochondrial ROS scavenger (MitoTEMPO) did not show enhanced cell survival, suggesting that cell death is not caused by the generation of ROS (Extended Data Fig. 1c,d).

Our hypothesis that alternative metabolites and FA metabolism provide an energy reserve for OXPHOS was supported by experiments, in which optic nerves were incubated for 16 h in glucose-free aCSF in combination with severe hypoxia (N_2_ atmosphere). Under hypoxia, cell death was extensive with 76% PI-labeled cells, suggesting that all cell types are affected (Fig. 1g). Thus, in the absence of glucose, virtually all glial cells appeared to have survived by OXPHOS.

We next asked directly whether FAs metabolized by β-oxidation comprise the postulated energy reserve. Optic nerves were incubated without glucose and under normoxia, but in the presence of 25 µM 4-bromocrotonic acid (4-Br), a nonspecific thiolase inhibitor of mitochondrial FA β-oxidation and ketolysis. Application of this drug dramatically reduced the rate of overall cell survival to 30% at 16 h (Fig. 1h). Importantly, 4-Br had no effect on glial survival in the presence of glucose (Fig. 1h), ruling out unspecific toxicity. Next, we tested 5 µM thioridazine (Thio), an inhibitor of peroxisomal β-oxidation that also claimed to block mitochondrial β-oxidation^18^. However, Thio had no obvious effect in the absence of glucose (Fig. 1i). This suggests that with respect to cell death peroxisomal β-oxidation is sufficiently compensated by mitochondrial β-oxidation (but see below for the effect on axonal conduction).

### Energy scarcity causes myelin thinning and vesicular demyelination

**Fig. 2:**
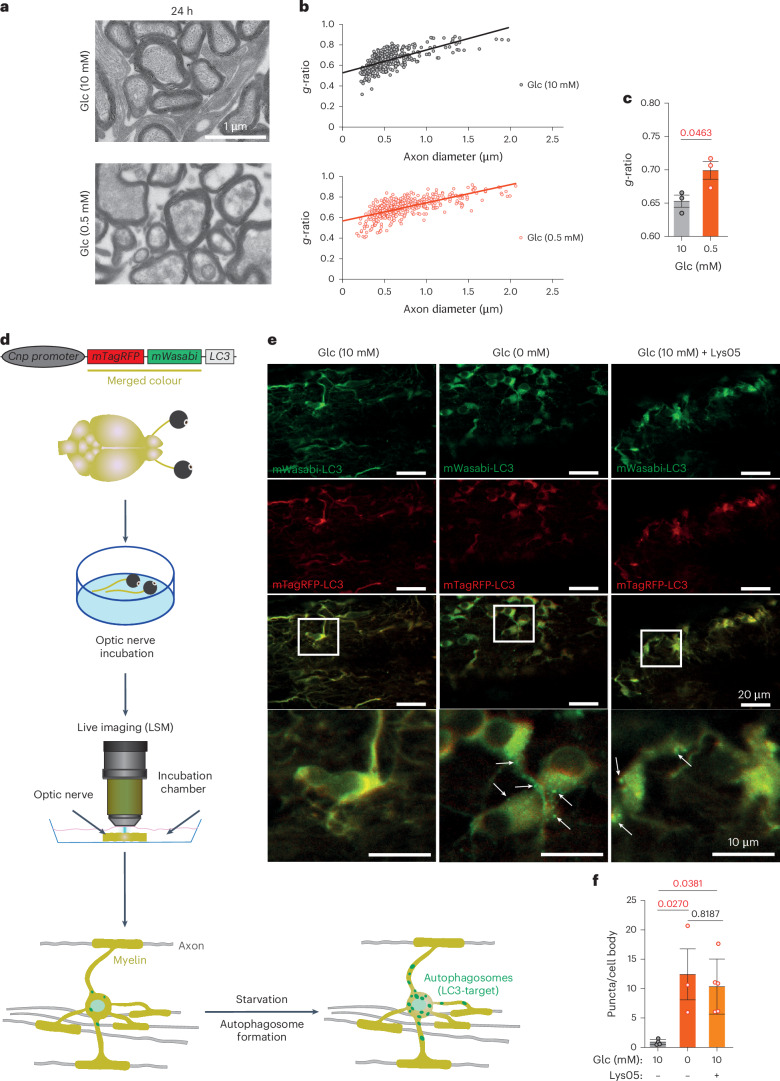
Glucose deprivation of the optic nerve induces autophagy and loss of myelin integrity. **a**, Electron microscopic images of optic nerve cross-sections (wild-type mice aged 2 months), taken after 24 h of incubation in medium with 10 mM glucose (top: *N* = *n* = 3) or 0.5 mM glucose (bottom: *N* = *n* = 3). **b**, Scatter plot of calculated *g*-ratios (outer fiber diameter/axon diameter) as a function of axon caliber, revealing myelin loss when optic nerves are exposed to low glucose (red dots, bottom) compared with 10 mM glucose-containing medium (black dots, top). **c**, Bar graph with calculated mean *g*-ratios (same data as in **b**) (*N* = *n* = 3 for both 0 mM and 10 mM; error bars: mean ± s.e.m.; unpaired, two-tailed Student’s *t*-test). **d**, Schematic depiction of the *pCNP-mTagRFP-mWasabi-LC3* transgene used for oligodendrocyte-specific expression of the LC3 fusion protein. The two fluorophores (red and green) give LC3 a yellow color and reveal diffuse cellular distribution. Note that mTagRFP-mWasabi-LC3 becomes an autophagosome-specific marker, when LC3 is translocated to the membrane of newly formed autophagophore/autophagosomes (green puncta). **e**, Live imaging of optic nerves (ex vivo) from a *mTagRFP-mWasabi-LC3* transgenic mouse, incubated in aCSF with 10 mM (left) or 0 mM (middle) glucose. Note the ubiquitous expression of the LC3 fusion protein. Specific labeling of autophagosomes (green puncta, arrows on image inset) occurs only in the absence of glucose with an all-or-none difference. Right, the accumulation of autophagosomes also in oligodendrocytes in the presence of 10 mM glucose (Glc) after applying the specific inhibitor, Lys05 (10 µM). **f**, Quantification of the data in **e**, normalized to the number of cell bodies (adult mice, aged 2–5 months; *N* = *n* = 4 for 10 mM Glc, *N* = *n* = 5 for 10 mM Glc + Lys05 and *N* = *n* = 3 for 0 mM Glc; error bars: mean ± s.e.m.; one-way ANOVA, Tukey’s test). Source data

**Extended Data Fig. 2:**
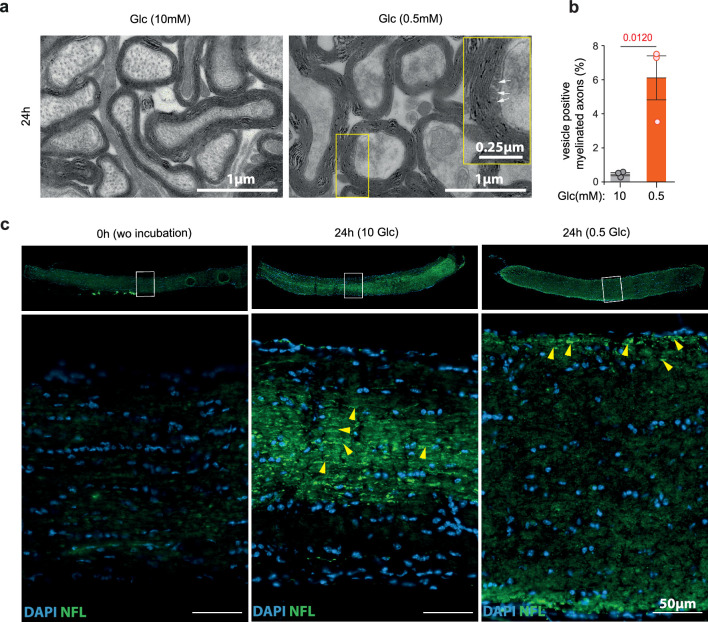
Vesicle formation in the myelin compartment is independent of ongoing axonal degeneration in metabolically stressed optic nerves. **a**, Electron micrographs of wildtype optic nerves (age 2 months), incubated for 24 in 10 mM glucose (left) or 0.5 mM glucose (right). Note periaxonal vesicular structures (white arrows) indicating myelin degradation under low glucose. **b**, Quantification of the data in (**a**) comparing percentage of myelinated axon cross sections with periaxonal vesicular structures (N = n = 3 for both conditions; mean ± SEM, unpaired two-tailed Welch’s t-test). **c**, longitudinal optic nerve section prepared freshly (0 h, left), after 24 h in 10 mM glucose (middle) or 0.5 mM glucose (right), immunostained for NF-L (green) and counter-stained with DAPI (blue). Yellow arrowheads point to degenerating axons (N = n = 3-4 for each condition). Source data

If FAs of the white matter were the main source of metabolic energy, starvation might even lead to a visible loss of myelin membranes. To directly determine this, we maintained optic nerves in low (0.5 mM) or regular (10 mM) glucose medium and analyzed them 24 h later (that is, before major glial cell death; Fig. 1c) by electron microscopy (EM) and quantitative morphometry (Fig. 2a–c). Starvation led to increased *g*-ratios (Fig. 2a–c). However, these numbers were not conclusive because acute starvation caused nonspecific swelling of the axonal and myelin compartments. Compatible with active demyelination was the emergence of vesicular structures underneath the myelin sheaths in starved nerves (Extended Data Fig. 2a,b), possibly caused by autophagy of myelin as reported^19^ (but see LC3^+^ puncta in oligodendrocyte somata further below). Most probably, the vesiculation of the innermost myelin layer is caused by the detachment of myelin basic protein (MBP)^20^.

When kept in 10 mM glucose for 24 h, optic nerve axons appeared morphologically normal by EM, whereas those at 0.5 mM glucose revealed loss of integrity of cytoskeletal elements (Fig. 2a). This raises the possibility that axonal degeneration rather than glucose deprivation causes myelin thinning. Although the dissection of axons will invariably cause their Wällerian degeneration, which is itself triggered by loss of NAD^+^ and ATP^21^, we also analyzed the ‘intact’ nerves for signs of axonal degeneration. As predicted, when immunostained for a proteolysis-dependent NF-L epitope, a very specific marker for neurodegeneration^22^, the molecular signature of Wällerian degeneration was also detected in nerves kept in 10 mM glucose for 24 h (Extended Data Fig. 2c). Thus, starvation rather than axonal degeneration triggers the observed demyelination.

### A basal level of autophagy in oligodendrocytes

**Extended Data Fig. 3:**
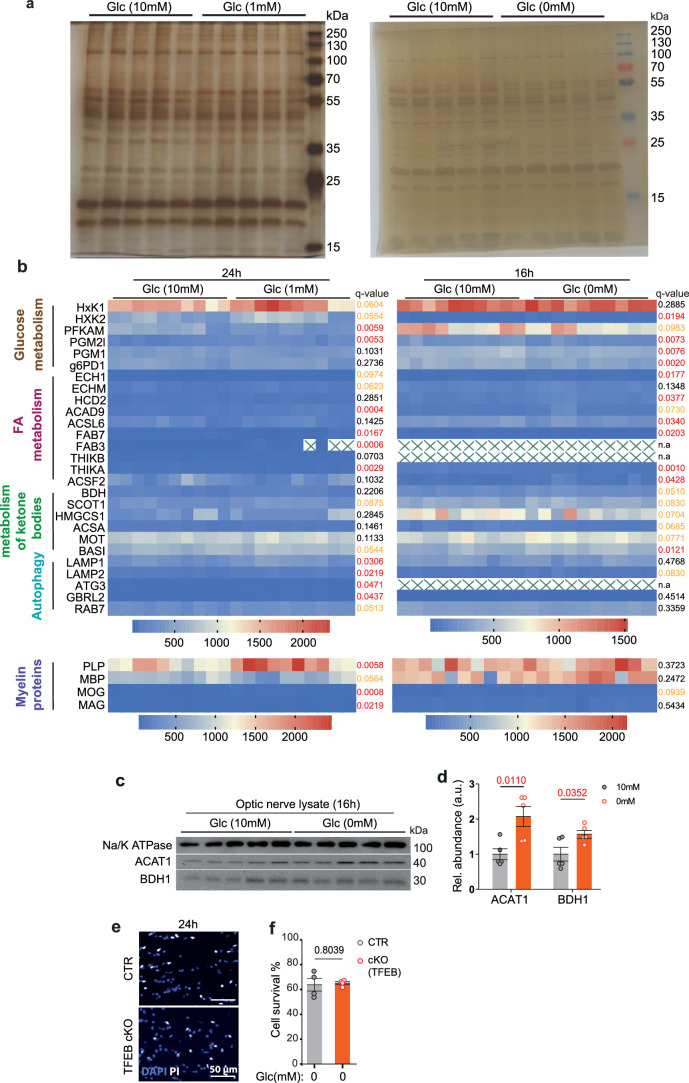
Increased autophagy and lipid metabolism in metabolically stressed optic nerves. **a**, Left: silver stained gels of optic nerve lysates from 2 month old wildtype mice (both genders), prepared after 24 h incubation in 10 mM glucose or 1 mM glucose (two nerves pooled per lane; one lane is equivalent to one sample). N = n = 5 for both conditions. Right: optic nerve lysates prepared after 16 h in 10 mM glucose or 0 mM glucose. Note the lack of major protein degradation. N = n = 5 for both conditions. **b**, Relative abundance of selected proteins in optic nerve lysates after 24 h in 1 mM glucose (left, N = n = 5) or 16 h in 0 mM glucose (right, N = n = 5). Note that enzymes of glucose and lipid metabolism show only moderate changes in abundance. Autophagy related proteins are increased in the presence of 1 mM glucose only, indicating a requirement of glucose for RNA synthesis and protein expression (N = n = 5, two technical replicates each; moderated t-statistics (more details in methods section)); Statistical significance (q-value) depicted on the right side of each panel. **c, d**, Western blots of lysates from wildtype optic nerves, incubated in 10 mM or 0 mM glucose for 16 h (age 8-12 weeks old, N = n = 5 for each condition) (**c**) and quantification of ACAT1 and BDH1 (**d**). Normalized to protein input (mean ± SEM, unpaired two-tailed t-test). **e**, Cell survival of 24 h glucose-deprived optic nerves from TFEB cKO mice (N = n = 4) and controls (N = n = 4; age 8-12 weeks). Images from longitudinal sections were stained with PI and DAPI. **f**, Quantified data from (**e**). There is no difference of cell survival (mean ± SEM, unpaired two-tailed Welch’s t-test). N and n indicate the total number of independent samples for each condition and the total number of independent experiments, respectively. Source data

We also subjected entire optic nerves to quantitative proteome analysis after either 16 h in glucose-free medium (when all cells survive) or 24 h in 1 mM glucose-containing medium, both in comparison to a regular medium (10 mM glucose). It is interesting that we noticed a minor trend toward higher myelin protein abundance (Extended Data Fig. 3a,b), perhaps because autophagy liberates proteins from compact myelin that are otherwise not solubilized. In these nerve lysates, the abundance of some glycolytic enzymes was reduced (for example, PFKAM (phosphofructokinase, muscle)) whereas fatty acid-binding proteins (FABP3) and enzymes of FA metabolism (acyl-COA dehydrogenase 9 (ACAD9)) were increased. A higher steady-state level of some autophagy-related proteins was detected, but only when nerves were maintained in low (1 mM) glucose (Extended Data Fig. 3b), most probably because some glucose is required for the pentose phosphate pathway, nucleotide synthesis and a transcriptional response. When assessed by EM (Fig. 2a–c), compact myelin appeared decreased, which would imply a greater loss of lipids than proteins. After 16 h in glucose-free medium, western blotting revealed a significant increase of acetyl-CoA acetyltransferase 1 (ACAT1) and 3-hydroxybutyrate dehydrogenase 1 (BDH1), enzymes involved in FA and ketone body metabolism, respectively (Extended Data Fig. 3c,d).

In adult mice, food withdrawal induces LC3^+^ autophagosomes in neuronal perikarya but not in axons^23^. Nevertheless, it is possible that myelin degradation is mediated by autophagy. To study autophagy in optic nerves, we generated a new line *of pCNP-mTagRFP-mWasabi-LC3* transgenic mice that express a tandem (pH-sensitive) fluorescent tag^24^ in oligodendrocytes (Fig. 2d). Indeed, 8.5 h after glucose withdrawal from optic nerves, we observed the accumulation of autophagosomes (Fig. 2e,f) in oligodendrocyte somata and processes as reported before^19^. In glucose-containing medium (10 mM), however, these organelles were detectable only when their degradation was specifically inhibited, for example, by Lys05 (Fig. 2e(right column),f). This suggests that in oligodendrocytes a basal level of autophagy always exists that increases only on glucose deprivation. This may explain why transcriptional upregulation of autophagy genes by its master regulator TFEB (transcription factor EB) is not essential for utilizing FAs from the myelin compartment (see also below for nerve recordings), as evidenced by the unaltered cell survival of glucose-deprived optic nerves from oligodendrocyte-specific TFEB conditional knockout (cKO) mice (Extended Data Fig. 3e–f).

### Oligodendrocyte lipid metabolism supports axonal function in starved optic nerves

**Fig. 3:**
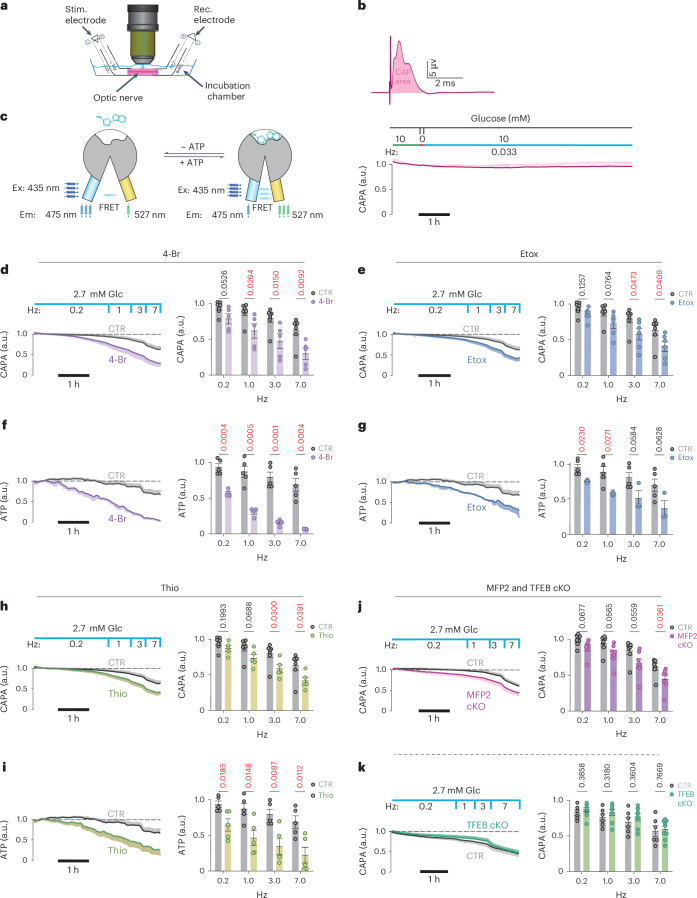
FA β-oxidation in oligodendrocytes supports axonal energy metabolism and function. **a**, Stimulating (Stim.) and recording (Rec.) CAPs from isolated optic nerves and monitoring axonal ATP by ratiometric FRET analysis. **b**, Top, typical CAP at 10 mM glucose with the CAPA shaded in red below. Bottom, recording of a stable CAPA, normalized to 1.0 (at 10 mM glucose, normoxia, low spiking rate (1 per 30 s)). Note a 5-min glucose withdrawal step to deplete astroglial glycogen. a.u., arbitrary units. **c**, Ratiometric FRET analysis using transgenically expressed^5^ ATP sensor ATeam1.03^YEMK^ (Ex and Em depict maximum excitation and emission wavelength respectively). **d**, Optic nerves, maintained functionally stable at 2 mM glucose and low spiking activity (0.2 Hz), exposed to 4-Br (25 µM; *N* = *n* = 5), an inhibitor of mitochondrial FA β-oxidation. Note the progressive decline of optic nerve conductivity (*N* = *n* = 7). **e**, Optic nerves exposed to Etox (5 µM, *N* = 6, *n* = 6), an inhibitor of long-chain FA uptake into mitochondria. Note the faster declining CAPA (*N* = *n* = 7). **f**, Same as in **d**, demonstrating a progressive loss of axonal ATP. Note the faster and stronger effect on the axonal ATP levels (*N* = *n* = 4) compared with controls (*N* = *n* = 5). **g**, Axonal ATP in Etox-treated nerves (*N* = *n* = 3) and controls (*N* = *n* = 5) as before. **h**, Optic nerves stimulated as before but in the presence of Thio (5 µM, *N* = 5, *n* = 5), an inhibitor of peroxisomal β-oxidation (*N* = *n* = 7). Note the difference to cell survival which is independent of peroxisomal β-oxidation (in Fig. 1i). **i**, Axonal ATP in Thio-treated nerves (*N* = *n* = 5) and controls (*N* = *n* = 5) as before. **j**, Optic nerves from *Cnp-Cre*^*+/-*^*::Mfp2*^*flox/flox*^ mice, lacking peroxisomal β-oxidation in oligodendrocytes^27^ and controls, at 2.7 mM glucose with increasing stimulation frequency (*N* = *n* = 7 each). Stronger CAPA decline in mutant nerves (7 Hz) confirms the role of oligodendrocytes in metabolic support. **k**, Optic nerves from *Cnp-Cre*^*+/−*^*::Tfeb*^*flox/flox*^ mice (*N* = *n* = 9) and controls (*N* = *n* = 6), showing that FA mobilization does not depend on de novo autophagy induction. All mice are aged 2 months (from both sexes). Bar graphs are mean ± s.e.m. (unpaired, two-tailed Student’s *t*-test) of data recorded in the last 5 min at each frequency. Controls are shared across **d**, **e** and **h**. Source data

As axonal conduction is energy dependent, we asked whether FA metabolism can support axon function under low glucose conditions. As a readout in acutely isolated optic nerves, we determined the size of the evoked compound action potential (CAP) after electrical stimulation^4^ (Fig. 3a,b). These recordings were performed in combination with real-time monitoring of the axonal ATP levels in the same nerves, using a genetically encoded ATP sensor expressed in the axonal compartment^5^ (Fig. 3c).

**Extended Data Fig. 4:**
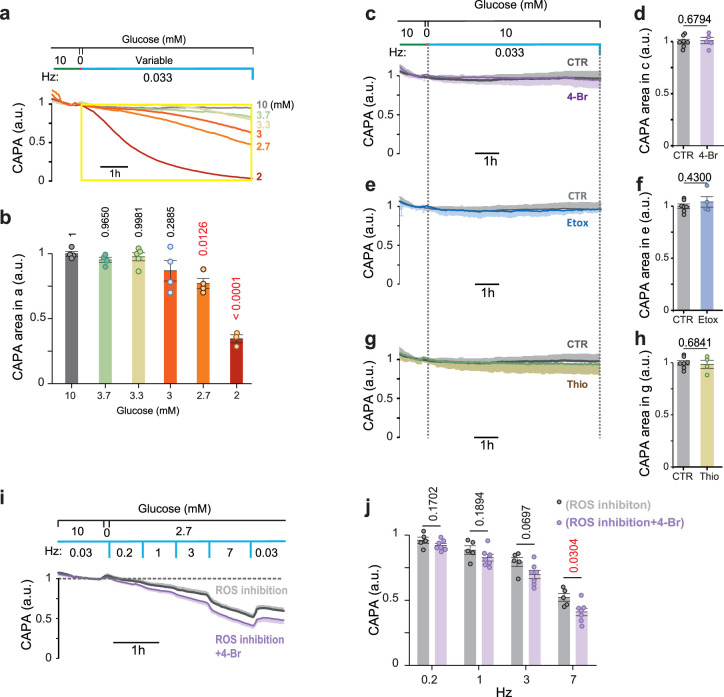
Decline in starved nerve function upon beta-oxidation inhibition is not caused by inhibitors cytotoxcicity or mitochondrial ROS generation. **a**, Empirical determination of the minimal glucose concentration at which optic nerves can spike at low frequency (1/30 s) for 3 hours without loss of compound action potential area (CAPA), normalized to control values with 10 mM glucose. **b**, Bar graphs of the CAPA summation (CAPA area) calculated for the time window (yellow box in **a**) for different glucose concentrations (N = n = 3 for 10, 3.7, 3 and 2.7 mM glucose condition, N = n = 3 for 2 mM and N = n = 5 for 3.3 mM glucose condition, one-way ANOVA, Tukey’s test). **c**, Recordings in aCSF+10 mM glucose ±25 μM 4-Br (N = n = 7 for control and N = n = 5 for 4-Br treated nerves). **d**, Bar graph of the CAPA summation for the defined time window (dashed lines) in (**c**). Values normalized to control. **e**, Normal conduction in aCSF with 10 mM glucose and 5 μM Etomoxir (Etox; N = n = 4) in comparison to 10 mM glucose control (same data as in **c**; N = n = 7). **f**, Bar graph comparing the calculated CAPA summation for the time window between the dash lines in (**e**). obtained values were normalized to control condition. **g**, Normal conduction in aCSF with 10 mM glucose and 5 μM Thioridazine (Thio; N = n = 4 in comparison to 10 mM glucose (same data as in **c**; N = n = 7)). **h**, Bar graph comparing the calculated CAPA summation for the depicted time window (dash lines) in (**g**). Obtained values were normalized to control. **i**, Conductivity under low glucose (aCSF+2.7 mM glucose +/-4-Br(25 μM)) in the presence of the ROS production inhibitor (S3qel-2, 10 μM) and mitochondrial ROS scavenger (MitoTEMPO, 10 μM). N = n = 5 for control (without 4-Br) and N = n = 7 (plus 4-Br). **j**, Bar graph comparing calculated CAPA from the data in (N &n the same as in (**i**)) for the average of CAPA recorded during the last 5 min of each step of the RAMP protocol. All nerves from wild type mice (age 8-12 weeks, from both genders) unless mutants specified. All error bars: mean ± SEM, unpaired two-tailed t-test, unless indicated. N and n indicate the total number of optic nerves for each condition and the total number of independent experiments, respectively. Source data

We first determined empirically the (set-up-specific) threshold level of glucose concentration (Extended Data Fig. 4a,b), at which acutely isolated optic nerves, after 5 min of glycogen depletion, remained sufficiently energized. This was defined as maintaining a low firing rate (0.2 Hz) for 2 h without decline of the CAP area (CAPA) (Fig. 3d; here: 2.7 mM glucose in aCSF). A subsequent gradual increase of the stimulation frequency (to 1 Hz, 3 Hz and 7 Hz) caused a gradual decline of the CAP, that is, an increasing fraction of axons with conduction blocks. Both preservation and decline of axon function could be quantified by calculating the curve integral, with the CAPAs plotted as a function of time. Similar to the cell survival experiments (Fig. 1h), we inhibited FA catabolism under starvation conditions. Importantly, when recordings were done in the presence of 4-Br, an inhibitor of thiolase (Fig. 3d) or etomoxir (Etox), an inhibitor of mitochondrial carnitine palmitoyltransferase 1 (CPT1) (Fig. 3e), these blockers of the mitochondrial FA β-oxidation caused a much more rapid decay of CAPA, that is, loss of conductivity. When applied in the presence of 10 mM glucose, these drugs had no toxic effects (Extended Data Fig. 4c–h). Under all conditions, we also monitored axonal ATP levels, which revealed strong parallels to the electrophysiological recordings (Fig. 3f,g). Thus, when glucose is limiting, the functional integrity of spiking axons is supported by FA degradation.

**Extended Data Fig. 5:**
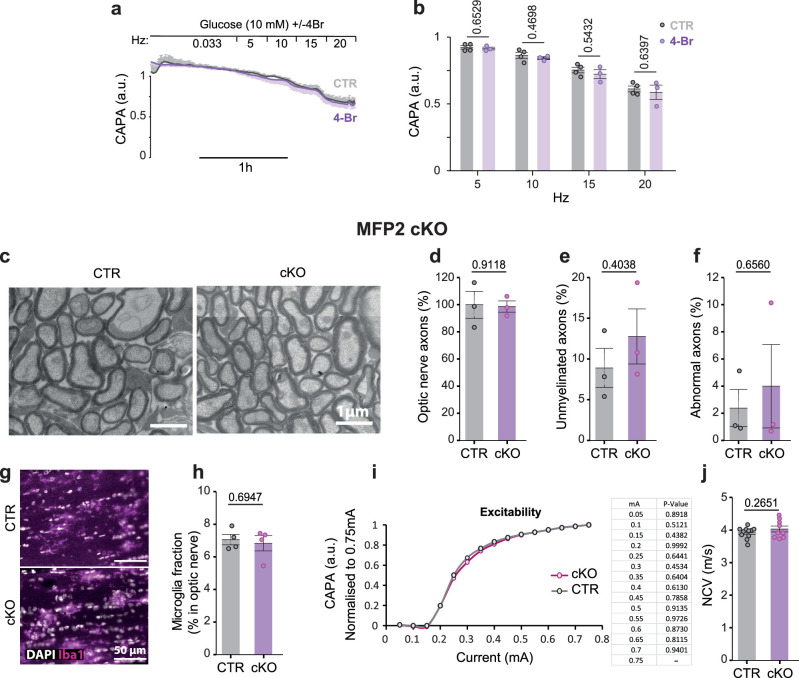
Inhibition of beta-oxidation in 10 mM glucose or peroxisomal beta-oxidation in oligodendrocytes does not affect optic nerves at histological and electrophysiological level at young adult mice. **a**, Spiking at high frequencies. Recordings were in aSCF (10 mM glucose) ± 25 μM 4-Br, using a RAMP protocol of increasing frequencies between 5 and 20 Hz (N = 3, n = 4 for control and N = 2, n = 3 for 4-Br treated nerves). **b**, Bar graphs of CAPA, calculated for each nerve over 5 min at the indicated frequencies (from **a**). **c**, EM images of MFP2 cKO optic nerves. **d**, Axon numbers in MFP2 cKO (N = 2, n = 3) versus controls (N = n = 3). **e**, Unmyelinated axon numbers (in **m**) in MFP2 cKO (N = 2, n = 3) versus controls (N = n = 3). **f**, Percentage of axons with abnormal morphology in MFP2 cKO (N = 2, n = 3) versus controls (N = n = 3). **g**, Longitudinal optic nerve section of MFP2 cKO and controls, immunostained for Iba1 (counterstained with DAPI). **h**, Number of Iba1+ microglia normalized to DAPI+ nuclei (N = n = 4 for control and MFP2 cKO). **i**, Normal excitability of optic nerves (N = 6, n = 11 for CTR and N = 4, n = 7 for MFP2 cKO). **j**, Optic nerve conduction velocity (NCV) in controls (N = 7, n = 11) and MFP2 cKO (N = 6, n = 9). All nerves from wild type mice (age 8-12 weeks, male and female) unless mutants specified. All error bars: mean ± SEM, unpaired two-tailed t-test. N and n indicate the total number of used mice for each condition and the total number of independently recorded/analyzed optic nerves, respectively. Source data

To confirm that the decline of the CAP, as observed under starvation, was not the result of ROS production, we repeated our recordings in the presence of ROS inhibitors and scavengers. Indeed, these drugs could not ‘rescue’ the CAP decline (Extended Data Fig. 4i,j). To also rule out the possibility that inhibition of β-oxidation interferes with degradation of toxic FAs generated in hyperactivated neurons^25^ leading to the decline of CAPs, we compared optic nerve conduction in the presence of 10 mM glucose. Indeed, high-frequency (5–20 Hz) conduction of these nerves remained the same in the presence or absence of 4-Br (Extended Data Fig. 5a,b).

Next, we applied 5 µM Thio, an inhibitor of peroxisomal β-oxidation. It is interesting that, and different from cell survival assays in the complete absence of glucose (Fig. 1i), in the electrophysiological experiments at low glucose similar results were obtained when inhibiting β-oxidation in mitochondria or peroxisomes (Fig. 3h,i). This may reflect the different energy requirements of basic survival and axonal conduction and also the extensive periaxonal localization of peroxisomes.

As our pharmacological treatments lacked cell-type specificity, we used genetics to selectively perturb β-oxidation in oligodendrocytes. Unfortunately, that was not possible for mitochondria, because even a triple-KO of all *CPT1* genes would not block uptake of short-/medium-chain FAs and lead to the accumulation of neurotoxic acyl-carnitine^26^. However, peroxisomal FA β-oxidation could be specifically targeted in *Cnp-Cre*^*+/−*^*::Mfp2*^*flox/flox*^ mutant mice^27^. Indeed, increasing the axonal spiking frequency in these mutant nerves caused the same increase in conduction blocks (that is, decrease of CAPA) as seen in Thio-treated nerves (Fig. 3j). In agreement with earlier studies, there were no underlying structural abnormalities of *Cnp-Cre*^*+/−*^*::Mfp2*^*flox/flox*^ optic nerves, including the number of axons, number of unmyelinated axons, number of ultrastructural axonal defects and number of microglia^27^. Also, basic electrophysiological properties of the nerves, including excitability and nerve conduction velocity, were unaltered (Extended Data Fig. 5c–j). This demonstrates directly a role for oligodendrocytes in the support of starving axons, with a important role for β-oxidation in peroxisomes, many of which reside in the myelin compartment^14^.

**Fig. 4:**
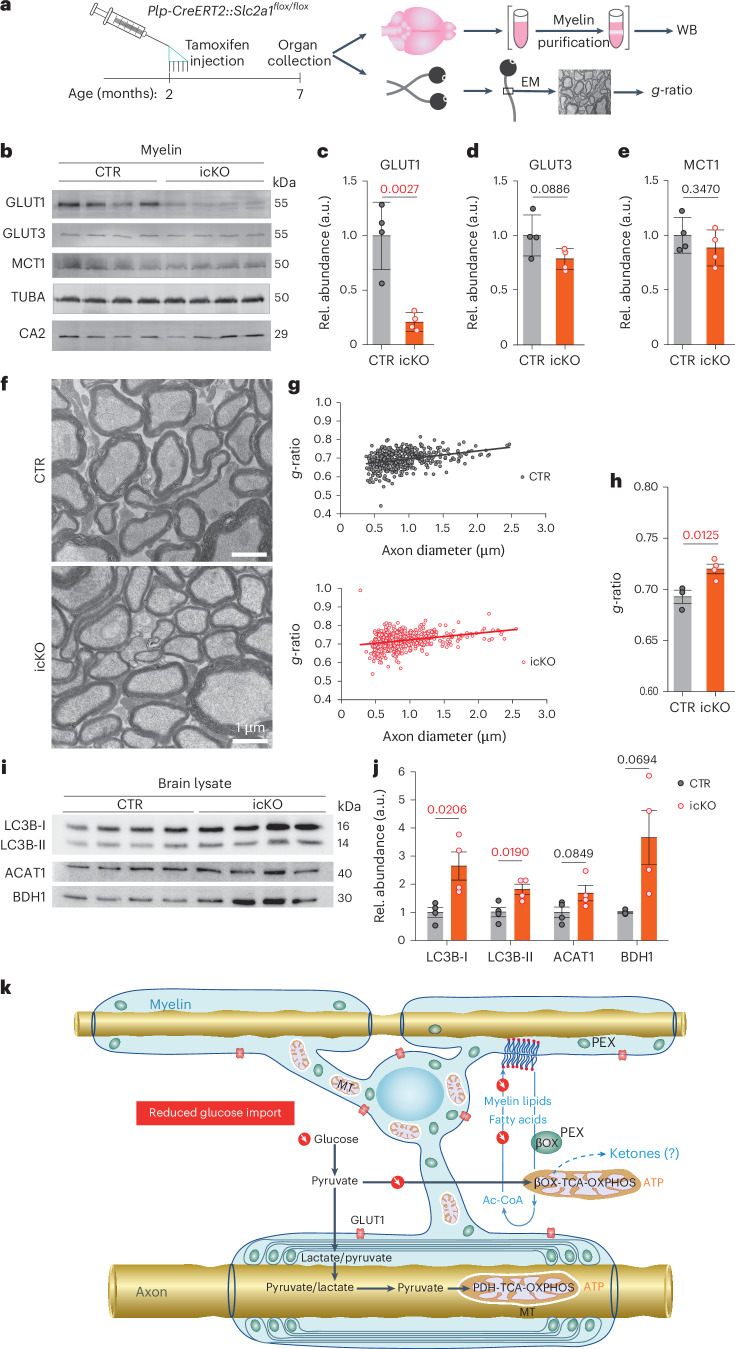
Oligodendroglial glucose starvation leads to a gradual myelin loss. **a**, Targeting GLUT1 expression in oligodendrocytes. *Plp-CreERT2::Slc2a1*^*flox/flox*^ mice received tamoxifen at age 2 months for phenotype analysis 5 months later. **b**, Western blot (WB) analysis of purified myelin membranes from whole-brain lysates. Note the decrease (quantified in **c**–**e**) of (oligodendroglial) GLUT1 (**c**), but not (neuronal) GLUT3 (**d**) or panglial MCT1 (**e**); CA2 (for control (CTR), *N* = 4; for icKO, *N* = 4; error bars: mean ± s.e.m., unpaired, two-tailed Student’s *t*-test). Rel., Relative. TUBA, α-tubulin. **f**, Electron micrographs of optic nerve cross-section from GLUT1 mutant icKO (*N* = 4; CTR: *N* = 4). Note the thinning of myelin in the absence of axonal degeneration. **g**, Scatter plot of calculated *g*-ratios (fiber diameter/axon diameter) from optic nerve EM data, with regression lines as a function of axon diameter. **h**, Myelin thinning in GLUT icKO mice (*N* = 4) compared with controls (*N* = 3). Error bars: mean ± s.e.m., unpaired, two-tailed Student’s *t*-test. **i**,**j**, Western blots of brain lysates from GLUT1 icKO mice (**i**) and quantification (**j**), normalized to protein input (fast green) (*N* = 4 for CTR and icKO; mean ± s.e.m., heteroscedastic for BDH1; Student’s *t*-test). **k**, Proposed working model of glycolytic oligodendrocytes with a myelin compartment that constitutes a lipid-based energy buffer. During normal myelin turnover, the degradation of myelin lipids in lysosomes liberates FAs for β-oxidation (β-Ox) in mitochondria (MT) and peroxisomes (PEX), leading to new myelin lipid synthesis. When glucose availability is reduced, as modeled in GLUT1 icKO mice, myelin synthesis drops and FA-derived acetyl-CoA begins, supporting mitochondrial respiration for oligodendroglial survival. This shift of normal myelin turnover to lipid-based ATP generation allows oligodendrocytes to share relatively more glucose-derived pyruvate/lactate with the axonal compartment to support ATP generation and prevent axon degeneration. Note that glucose is never absent in vivo and that myelin-associated peroxisomes^14^ are better positioned than mitochondria to support axons with the products of FA β-oxidation. Whether oligodendrocytes also use ketogenesis to metabolically support axons and other cells is not known. Source data

**Extended Data Fig. 6:**
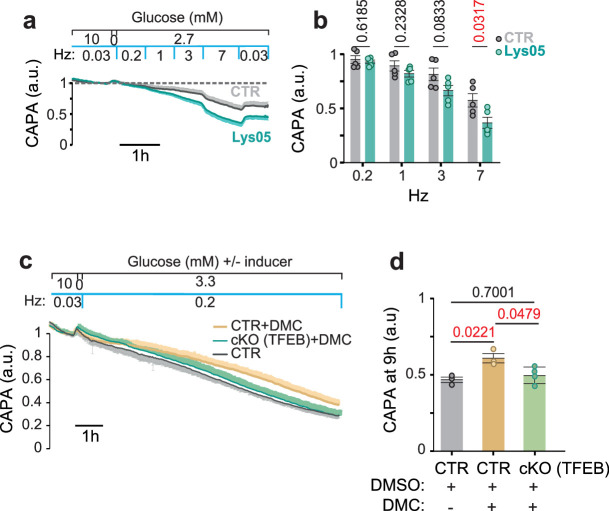
Preexisting autophagy contributes to oligodendroglial support of axonal conduction in starved optic nerves. **a**, CAP recordings from wild type optic nerves kept under low glucose condition (aCSF with 2.7 mM glucose) in the presence or absence of 10 μM autophagy inhibitor Lys05 (8-12 weeks old, male and female; N = n = 5 each condition). **b**, Bar graph representing the average CAPA during the last 5 min of each stimulation step (same data in **a**; mean ± SEM, unpaired two-tailed t-test). **c**, Effect of the autophagy inducer DMC (40 μM) on nerve conduction under low glucose condition. Note that the inducer improves CAPA in wildtype optic nerves but in nerves from TFEB cKO mice. **d**, Quantification of the data in (**c**) with a comparison of CAPA at 9 h (average of 5 min recordings). Statistics: N = n = 3 for control ( + DMSO), N = n = 3 for control +DMC, and N = n = 4 for TFEB + DMC, 3-5 months old mice from both genders, (mean ± SEM, one-way ANOVA, Tukey’s test). Source data

We further investigated whether autophagy plays a role in myelin turnover and FA metabolism under starvation conditions. TFEB activates autophagy-related genes and lysosomal functions^28^ also in oligodendrocyte lineage cells^29^. We generated *Cnp-Cre::TFEB*^*flox/flox*^ mice for oligodendrocyte-specific ablation, but found that mutant optic nerve conduction remains indistinguishable from wild-type nerves with respect to the ex vivo CAP decline (Fig. 3k) and conduction velocity. In contrast, application of the autophagy inhibitor Lys05 to wild-type nerves under limiting glucose concentrations caused a faster CAP decline (Extended Data Fig. 6a,b). This suggests a contribution of pre-existing autophagy to axonal energy metabolism. In turn, the autophagy inducer 3,4‐dimethoxychalcone (DMC), an activator of TFEB and TFE3, improved nerve function, but failed to do so in nerves from *Cnp-Cre::TFEB*^*flox/flox*^ mice (Extended Data Fig. 6c,d). Taken together, these observations suggest that autophagy pre-exists in oligodendrocytes and that the master regulator TFEB is not critical for the utilization of FAs in energy metabolism. We also detected the upregulation of autophagy in brain lysates of mice with reduced glucose availability to oligodendrocytes (see below; Fig. 4i,j).

### Ablating GLUT1 from mature oligodendrocytes in vivo reduces myelin thickness

Acute glucose deprivation of white matter in short-term ex vivo experiments showed proof of principle for the role of FAs as energy reserves. However, this model differs from long-lasting, chronic hypoglycemia, which can occur in real life, for example, on starvation. Starvation experiments are obviously not possible and have difficulty controlling side effects, such as ketosis and gluconeogenesis. To circumvent these and to only ‘glucose starve’ oligodendrocytes, we generated a line of tamoxifen-inducible *Plp1*^*CreERT2/+*^*::Slc2a1*^*flox/flox*^ conditional mutant mice. These lack glucose transporter 1 (GLUT1) expression specifically in mature oligodendrocytes after tamoxifen administration at the age of 2 months (Fig. 4a). We expected only a slow decline of glucose import, because GLUT1 is associated with myelin^4^ and should have a slow turnover similar to myelin structural proteins. Moreover, mutant oligodendrocytes remain gap junction coupled to astrocytes and *Plp1*^*CreERT2*^ recombination efficacy is not 100%. When testing purified myelin by western blot analysis 5 months after tamoxifen administration, we determined a significant but still incomplete decrease of GLUT1. In contrast, GLUT3, monocarboxylate transporter 1 (MCT1), α-tubulin and oligodendroglial carbonic anhydrase 2 (CA2) were unaltered in abundance (Fig. 4b–e).

**Extended Data Fig. 7:**
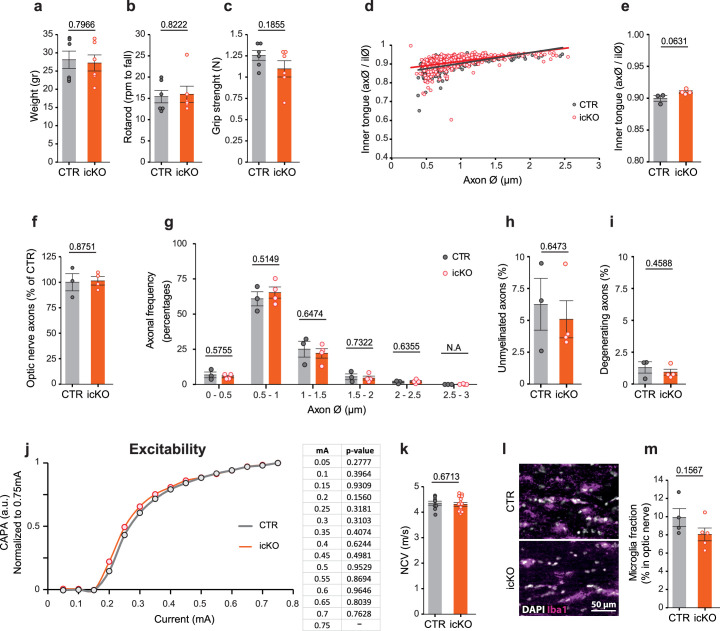
Hypomyelinated optic nerves in normally behaving GLUT1 icKO mice lack histological signs of pathology or gliosis. **a**, Normal body weight of GLUT1 icKO mice at the age of 7 months (5 months post tamoxifen). (N = 6, CTR and icKO). **b**, Normal rotarod performance of GLUT1 icKO mice shown as the speed (rpm) at which the mice fall (same mice in **a**). **c**, Normal forelimb grip strength of GLUT1 icKO mice (same mice as in **a**). **d**, Quantification of the inner tongue size as a function of axon caliber, by plotting the axon diameter (ax) by the respective diameter of a circle defined by the inner surface of the compacted myelin sheath (il), analogous to g-ratios (same images in Fig. 4f). **e**, Tendency for smaller average inner tongue sizes in GLUT1 icKO mice. (same data as in **d**; CTR, N = n = 3; icKO, N = n = 4). **f**, Normal number (density) of optic nerve axons in GLUT1 icKo mice normalized to controls (same images from Fig. 4f). **g**, Normal axon size distribution in optic nerve from GLUT1 icKo mice (same images from Fig. 4f; CTR, N = n = 3; icKO mice, N = n = 4). **h**, Normal percentage of unmyelinated axons in GLUT1 icKO mice (same images from Fig. 4f). **i**, Normal percentage of axons showing morphological abnormalities by EM analysis (from images in Fig. 4f). **j**, Normal excitability of optic nerves from GLUT1 icKO mice, recorded with increasing current of stimulation. Calculated CAPA for each current was normalized to recorded CAPA at 0.75 mA (CTR, N = n = 9; icKO, N = n = 15). **k**, Normal optic nerve conduction velocity (NCV) of GLUT1 icKO mice, calculated by dividing latency of the second CAP peak to the length of the nerve (common data with **j**; CTR, N = n = 6; icKO, N = n = 12). **l**, Longitudinal sections of GLUT1 icKO optic nerves immunostained for Iba1 and counterstained with DAPI. **m**, Normal number of Iba1+ microglia in optic nerve from GLUT1 icKO mice, normalized to the number of DAPI+ nuclei in the same area (same images in **l**). CTR N = 3, n = 4, icKO N = n = 5. All animals (both genders) were analyzed 4-5 months post tamoxifen injections. All error bars: mean ± SEM, unpaired two-tailed t-test. CTR, Control. Source data

**Extended Data Fig. 8:**
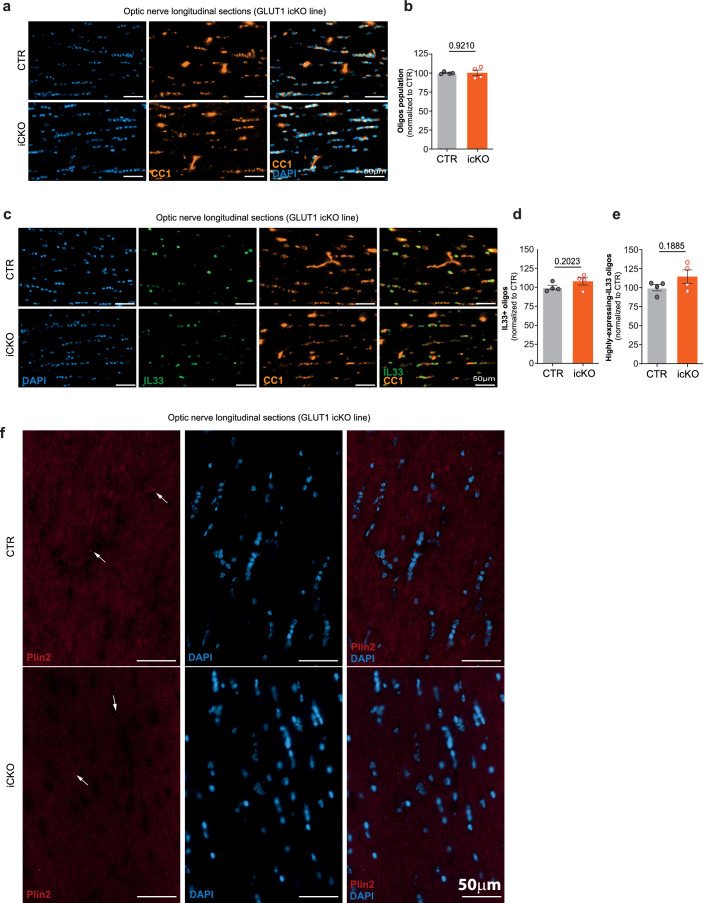
Optic nerves in GLUT1 icKO mice lack histological signs of changes in inflammatory/oligodendrocyte population or lipid droplets. **a**, Longitudinal sections of GLUT1 icKO optic nerves immunostained for CC1 (orange) and counterstained with DAPI (blue). **b**, Oligodendrocytes population in optic nerve from GLUT1 icKO mice calculated by dividing the DAPI spots positive for CC1 to total number of DAPI+ nuclei (DAPI + CC1 + /DAPI + ) in the same area. Data points were normalized to the mean of CTR; N = 4 for both CTR and icKO. **c**, Longitudinal sections of GLUT1 icKO optic nerves immunostained for CC1 (orange), IL-33 (green) and counterstained with DAPI (blue). **d-e**, Bar graphs showing percentage of oligodendrocytes were positive for inflammatory marker, IL-33, (CC1 + IL-33 + ) divided to total number of oligodendrocytes (DAPI + CC1 + ) (**d**) and percentage of oligodendrocytes exhibiting high expression of inflammatory marker, IL-33 (**e**). (data points were normalized to the mean of CTR; N = 4 for both CTR and icKO). **f**, GLUT1 icKO optic nerve longitudinal sections were immunostained for Plin2 (red) and counterstained with DAPI (blue). Note lipid droplets are barely detectable in optic nerve cells (N = 3 for both control and icKO nerves). All animals (from both genders) were analyzed at the age of 6-7 months (4-5 months post tamoxifen). An unpaired two-tailed t-test was performed for comparing different groups. Error bars indicate mean ± SEM, and individual data points displayed. CTR, Control. Source data

Importantly, *Slc2a1* conditional mutants from both sexes showed no obvious behavioral defects and lacked visible neuropathological changes (Extended Data Fig. 7a–c). However, when the myelin sheath thickness was quantified by EM (Fig. 4f), *g*-ratio analysis of the optic nerve revealed significant loss of myelin membranes in the absence of obvious axonal pathology (Fig. 4g,h and Extended Data Fig. 7d–i). There were also no signs of inflammation or altered electrophysiological properties (Extended Data Fig. 7j–m). In addition, CC1 and Plin2 immunostaining failed to show oligodendrocyte loss or the abnormal formation of lipid droplets in the optic nerve, respectively (Extended Data Fig. 8a,b,f). Whether the fraction of the recently identified immune oligodendrocytes^30^ changes on chronic starvation awaits a more detailed single-nucleus RNA sequencing analysis, but the number of oligodendrocytes expressing the relevant marker gene *IL33* was not increased (Extended Data Fig. 8c–e). Western blot analysis of mutant brain lysates showed elevated levels of LC3b I and II and BDH1, and a tendency for more mitochondrial ACAT1 (Fig. 4i,j). These data demonstrate that myelin metabolism continues when oligodendrocytes lack normal glucose uptake, suggesting that the mechanism of myelin loss during starvation is the ongoing catabolic arm of myelin turnover.

## Discussion

Our in vitro and in vivo data, when combined, led to a model of myelin dynamics that extends the model of glycolytic oligodendrocytes delivering metabolic support to fast spiking axons^2–5^ (Fig. 4k). Our key experiment was the direct analysis of both axonal conductivity and ATP levels in myelinated optic nerves under defined metabolic conditions, including low glucose, and in the presence of specific metabolic inhibitors, complemented by oligodendrocyte-specific, gene-targeting experiments in vivo. The latter allowed us to detect even a gradual loss of myelin membranes when oligodendrocytes have reduced glucose availability. Distinguishing the contribution of mitochondrial and peroxisomal β-oxidation in vivo remains difficult, but, regardless of the subcellular origin of FAs, membrane lipids are in constant horizontal flux between compartments, which also includes, for oligodendrocytes, myelin membranes that emerge as a large ‘lipid store’.

Myelin sheaths are wrapped within hours and days^31,32^ and continue to turn over in adult life when myelin synthesis and degradation are in equilibrium^11,33^. The anabolic arm of myelin membrane synthesis is well known and has been studied in the context of developmental myelination by the impact of nutritional deprivation. Studies in undernourished newborn rats were first performed in the late 1940s and in the following decades by radiolabeling studies. These data have shown the suppression of myelin synthesis under caloric restriction, which can be rescued by refeeding^34^. We propose likewise that, in our system, continued myelin synthesis comes to a halt on glucose deprivation.

However, the catabolic arm of myelin turnover^35^, which includes myelin degradation and FA breakdown, continues. This was shown directly in adult mice, in which the tamoxifen-induced loss of MBP, a protein required for myelin membrane incorporation, causes demyelination^11^. Importantly, after energy deprivation, mitochondrial FA β-oxidation can feed acetyl-coA without temporal delay into the tricarboxylic acid cycle and OXPHOS. In fact, this would be the fastest utilization of readily available metabolic energy. We note that, under real-life conditions, there can be transient hypoglycemia but no aglycemia. Thus, oligodendroglial FAs must only partially compensate for glucose, most probably sparing available glycolysis products for anaplerotic reactions or export and axonal support.

Alternatively, oligodendrocytes may use FA-derived acetyl-CoA to generate ketone bodies in the cholesterol pathway (by 3-hydroxy-3-methylglutaryl-CoA lyase or direct deacylation of acetoacetyl-CoA)^36,37^. This would match recent findings in *Drosophila* spp., where glycolytically impaired glial cells use FA β-oxidation in combination with ketogenesis to support neuronal metabolism^9,38^. In mammalian brains, ketone bodies increase with age^39^ and can spread horizontally^40^ such as pyruvate or lactate through the monocarboxylate transporter MCT1. All these metabolites can also pass via gap junctions to other glial cells in the ‘panglial’ syncytium^41^. Horizontal flux of FAs may also lead to their β-oxidation in other glial cells. Mice with cell-type-specific deletions of MCT1 (ref. ^42^), connexins and pannexins will help define these pathways in the future. The higher vulnerability of astrocytes to glucose deprivation is thus puzzling and may reflect an irreversible metabolic switch to glycolysis and therefore glucose dependency. Our experiments with specific inhibitors suggest that the toxic effects of ROS, as reported for astrocytes^43^, are less likely.

Unlike glycogen mobilization by astrocytes, FA β-oxidation by oligodendrocytes (or ketone body supplementation) cannot support rapid axonal firing, even for a short time^44^. Thus, on severe hypoglycemia, conduction blocks appear unavoidable once glycogen stores have been depleted^45^. However, in the absence of axonal spiking, oligodendroglial FA metabolism might suffice to prevent a more severe ATP decline that leads to irreversible axon loss^46^.

A limitation of our study is the lack of direct in vivo evidence that oligodendroglial β-oxidation supports axon function and survival under real starvation conditions. Starvation experiments are illegal and real hypoglycemia (also insulin induced) would initially introduce physiological responses (gluconeogenesis, ketogenesis) that interfere with and mask the paradigm itself. However, nature provides supporting evidence: hibernating animals have severely reduced blood glucose levels over a period of months^47,48^, but lack obvious neurodegeneration. It is interesting that, after hibernation, Syrian hamsters exhibit substantial changes of myelin lipids, with phospholipids but not cholesterol being lost^49^.

We note reports of white matter lesions being detectable by magnetic resonance imaging in patients in diabetic hypoglycemic coma^50^ or in severe anorexia nervosa^51,52^, which has been mechanistically unexplained. Thus, if nutritional stress is prolonged, the lack of normal myelin synthesis despite continued FA catabolism by oligodendroglia becomes macroscopically visible. Also the peripheral nervous system is involved because individuals with obesity who underwent gastric bypass (bariatric) surgery develop encephalopathy and peripheral neuropathy^53^. Similarly, physically starved rats^54^ showed peripheral demyelination by Schwann cells that share functions with oligodendrocytes in myelin lipid metabolism^2,17,55^.

Our findings have relevance for human neurodegenerative diseases. In pathological conditions with chronic hypometabolism, lack of normal myelin synthesis in oligodendrocytes (but continued FA catabolism) should become macroscopically visible, which may be the case in small-vessel disease. Also, in multiple sclerosis, axon degeneration has been attributed to energy failure^56^ which could by itself contribute to demyelination. Many neuropsychiatric diseases, including Alzheimer’s disease, have been associated with hypometabolism^17^ and white matter abnormalities^57–59^ or unexplained myelin abnormalities^60,61^. Long axons in the white matter are clearly a bottleneck of neuronal integrity. In a prolonged metabolic crisis, maintaining the oligodendrogial FA metabolism possibly being the key to prevent irreversible axon degeneration.

## Methods

### Animals

All mice were bred on a C57BL/6 background (except Aldh1l1-GFP) and kept under a 12 h:12 h day:night cycle with free access to food and water (temperature of 22 °C, 30–70% humidity). Experimental procedures were approved and performed in accordance with Niedersächsisches Landesamt für Verbraucherschutz und Lebensmittelsicherheit (LAVES; license no. 18/2962).

Transgenic mice were generated in-house by routine procedures, as previously described^14^. To visualize autophagosome in oligodendrocytes, a *mTagRFP-mWasabi-LC3* construct^24^ was placed under the control of the *Cnp* promoter. Genotyping was with forward (5′-CAAATAAAGCAATAGCATCACA-3′) and reverse (5′-GCAGCATCCAACCAAAATCCCGG-3′) primers, using the following PCR program: (30× 58 °C: 30 sec; 72 °C: 45 sec; 95 °C: 30 sec).

Eight other mouse lines were genotyped as previously published: (1) *Aldh1l1-GFP* for labeling astrocytes^62^; (2) *Cxcr-GFP* for microglia^63^; (3) *Cnp-mEos2* for oligodendrocytes^14^; (4) *Ng2-YFP* for OPC^64^; (5) *Mfp2*^*flox/flox*^*::Cnp-Cre*, targeting peroxisomal β-oxidation in myelinating glia^27,65^; (6) *Slc2a1*^*flox/flox*^*::Plp1*^*CreERT2*^, targeting GLUT1 in myelinating glia^66,67^; (7) *Tfeb*^*flox/flox*^*::Cnp-Cre*, targeting autophagy in myelinating glia^28,65^; and (8) *Thy1-Ateam*, encoding a neuronal ATP sensor^5^.

The following control genotypes were used in combination with the corresponding homozygous mutants: *Cnp*^*+/+*^*::Mfp2*^*flox/flox*^, *Slc2a1*^*flox/flox*^ + tamoxifen and *Cnp*^*+/+*^*::Tfeb*^*flox/ flox*^.

### Materials

Reagents were purchased from Merck unless otherwise stated.

### Artificial CSF solution for optic nerve incubation and recording

Optic nerve incubation and electrophysiological recordings were done under constant superfusion with aCSF containing (in mM): 124 NaCl, 23 NaHCO_3_, 3 KCl, 2 MgSO_4_, 1.25 NaH_2_PO_4_ and 2 CaCl_2_. The aCSF was constantly bubbled with carbogen (95% O_2_, 5% CO_2_). A concentration of 10 mM glucose (Sigma-Aldrich, ≥99%) was used as the standard (control). Incubation experiments were done with 0 mM glucose (‘glucose free’). For EM and proteomics experiments, 0.5 mM and 1 mM glucose (‘starvation’) were applied, respectively. For electrophysiological recordings, 2.7 mM glucose was applied as a starvation condition unless otherwise stated. Whenever a lower glucose concentration than 10 mM was applied, the difference was substituted by sucrose (which cannot be metabolized) to maintain osmolarity.

Specific inhibitors for—(1) mitochondrial β-oxidation, 4-Br^68^ (TCI, ≥98%); (2) peroxisomal β-oxidation, Thio (Sigma-Aldrich, ≥99%); (3) mitochondrial β-oxidation of long-chain FAs, Etox^69^ (Tocris, ≥98%); (4) ROS inhibitor, S3QEL-2 (ref. ^70^) (Sigma-Aldrich); (5) ROS scavenger, MitoTEMPO^71^ (Sigma-Aldrich); and (6) autophagy inhibitor, Lys05 (ref. ^72^) (Sigma-Aldrich, ≥98%)—were prepared freshly and added to the aCSF at a concentration of 25, 5, 5, 10, 10 and 10 μM, respectively. To block mitochondrial OXPHOS, 5 mM sodium azide was added to aCSF containing 0 mM glucose and 119 mM NaCl. The autophagy inducer, DMC^73^ (AdipoGen), was applied at 40 μM.

### Mouse optic nerve preparation and incubation

After cervical dislocation, optic nerves were dissected before the optic chiasma and each nerve were gently removed. The prepared nerves (attached to the eyeball) were transferred into a six-well plate containing 10 ml of aCSF adjusted to 37 °C. Another 90 ml of aCSF was circulating during the incubation period. To study the effects of anoxia, aCSF was bubbled with nitrogen (95% N_2_, 5% CO_2_; Air Liquide) instead of carbogen (95% O_2_, 5% CO_2_). To minimize the diffusion of oxygen into aCSF, the wells were sealed with parafilm.

### Determining glial cell survival

To label dying cells, optic nerves were exposed to PI (12 µM; Sigma-Aldrich) during the last hour of incubation. Nerves were subsequently washed (10 min) in 7 ml of aCSF. After 1 h fixation with 4% paraformaldehyde (PFA in 0.1 M phosphate buffer), the nerves were detached from the eyeball and frozen blocks were prepared in Tissue-Tek O.C.T compound (SAKURA). Sections (8 μm) were obtained by cryosectioning (Leica) and kept in the dark at −20 °C until further staining. Sections were washed in phosphate-buffered saline (PBS, 10 min) and stained with DAPI (1:20,000 of 1 mg per ml of stock), washed again in PBS (2× for 5 min) and mounted.

All images were taken with an inverted epifluorescent microscope (Zeiss Axio Observer Z1). Illumination and exposure time settings for PI and DAPI were kept constant for all images. For the fluorescent reporter lines, different exposure times were used, according to the observed signal intensity of each fluorophore. Sections (2–3) of optic nerves were imaged and tiled arrays were stitched by the microscope software (Zen, Zeiss) for quantification.

To determine the percentage of dying cells, Fiji software and Imaris software (v.8.1.2) were used. Stitched images were loaded in Fiji to trim areas of the optic nerves that contain dying cells unrelated to the experiment (that is, resulting from normal handling). After adjusting the thresholds for each channel, single cells were automatically marked over the nucleus, manually double-checked and corrected. In the last step, co-localization of signals was calculated and data were exported (Excel files) for statistical analysis. The percentage of dying cells was obtained by dividing the number of PI over DAPI^+^ nuclei (PI/DAPI). For determining the frequency of each cell type, the number of fluorescent cells (green fluorescent protein (GFP), yellow FP (YFP) or monomeric fluorescent protein Eos 2 (mEOS2)) was divided by the number of DAPI^+^ cells. To calculate the relative survival of each cell type, the percentage of each fluorophore-positive cell type was determined (astrocytes: *Aldh1l1*-GFP; microglia: *Cxcr1*-GFP; OPCs: *NG2*-YFP; oligodendrocytes: *Cnp*-mEOS2) after the corresponding PI/DAPI^+^ cells had been subtracted. The obtained values were normalized to the results for control conditions (10 mM glucose) and expressed as ‘cell survival rate’. To minimize the effect of signal intensity differences between different experiments, we adjusted the threshold for quantifications based on respective controls in each experiment. As we had dramatic cell death in starved nerves, blinding was not applicable.

### Myelin preparation

GLUT1-inducible cKO (icKO) mice were sacrificed 5 months after tamoxifen injection (age 7 months). A light-weight membrane fraction enriched in myelin was obtained from frozen half-brains, using a sucrose density gradient centrifugation as previously described^74^. Briefly, after homogenizing the brains in 0.32 M sucrose solution containing protease inhibitor (cOmplete, Roche), a crude myelin fraction was obtained by density gradient centrifugation over a 0.85 M sucrose cushion. After washing and two osmotic shocks, the final myelin fraction was purified by sucrose gradient centrifugation. Myelin fractions were washed, suspended in Tris-buffered saline (137 mM NaCl, 20 mM Tris-HCl, pH 7.4, 4 °C) and supplemented with protease inhibitor (Roche).

### Western blotting

Western blotting and Fast Green staining were performed as previously described^75^ using the following primary and secondary antibodies: ACAT1 (1:3,000, cat. no. 16215-1-AP, Proteintech), BDH1 (1:500, cat. no. 15417-1-AP, Proteintech), LC3B (1:2,000, cat. no. NB100-2220, Novusbio), Na^+^/K^+^ ATPase α1 (1:1,000, cat. no. ab7671, Abcam), GLUT1 (1:1,000)^76^, GLUT2 (1:1,000, cat. no. ab54460, abcam), GLUT3 (1:1,000, cat. no. ab191071, abcam), GLUT4 (1:1,000, cat. no. 07-1404, Millipore), MCT1 (1:1,000)^77^, CA2 (1:1,000)^78^ and α-tubulin (1:1,000, cat. no. T5168, Sigma-Aldrich), mouse immunoglobulin G heavy and light (IgG H&L) Antibody Dylight 680 Conjugated (1:10,000, cat. no. 610-144-002); rabbit IgG H&L Antibody DyLight 800 Conjugated (1:10,000, cat. no. 611-145-002, Rockland); horseradish peroxidase-conjugated secondary antibodies (1:5,000, cat. nos. 115-03-003 and 111-035-003, Dianova). Signal intensities, analyzed with the Image Studio software Licor or Fiji, were normalized to the corresponding total protein load, which was quantified by Fast Green staining. Obtained values were normalized to the mean of the respective values from control mice.

### Proteome analysis and western blots

Optic nerves were collected after incubation in aCSF with 10 mM glucose (control), 0 mM or 1 mM glucose, transferred to microtubes and kept at −80 °C until further analysis. To minimize variability, one nerve from a mouse was incubated under starvation or glucose-deprivation conditions and the other under control conditions. Nerves from two mice were pooled for protein extraction, homogenized in 70 μl of radioimmunoprecipitation analysis buffer (50 mM Tris-HCl; Sigma-Aldrich), sodium deoxycholate (0.5%; Sigma-Aldrich), NaCl (150 mM), sodium dodecylsulfate (SDS) (0.1%; Serva), Triton X-100 (1%; Sigma-Aldrich), EDTA (1 mM) and complete protease inhibitor cocktail (Roche) by using ceramic beads in a Precellys homogenizer (for 3× 10 s at 6,500 rpm) (Precellys 24, Bertin Instruments). After a 5-min centrifugation at 15,626*g* and 4 °C, the supernatant was used for protein determination (DC Protein Assay reagents, BioRad) according to the manufacturer’s protocol, with the absorbance of samples at 736 nm (Eon microplate spectrophotometer, Biotek Instruments). Proteins (0.5 µg) were separated on 12% SDS–polyacrylamide gel electrophoresis gels and subjected to silver staining^79^.

### Proteomics

Proteome analysis of purified myelin was performed as recently described^74,75^ and adapted to optic nerve lysates^11^. Briefly, supernatant fractions corresponding to 10 μg of protein were dissolved in lysis buffer (1% ASB-14, 7 M urea, 2 M thiourea, 10 mM dithiothreitol and 0.1 M Tris, pH 8.5). After removal of the detergents and protein alkylation, proteins were digested overnight at 37 °C with 400 ng of trypsin. Tryptic peptides were directly subjected to liquid chromatography–tandem mass spectrometry (LC–MS/MS) analysis. For quantification according to the TOP3 approach^80^, aliquots were spiked with 10 fmol μl^−1^ of Hi3 *E. coli* Standard (Waters Corp.), containing a set of quantified synthetic peptides derived from *Escherichia coli*. Peptide separation by nanoscale reversed-phase ultraperformance LC was performed on a nanoAcquity system (Waters Corp.) as described^11^. MS analysis on a quadrupole time-of-flight mass spectrometer with ion mobility option (Synapt G2-S, Waters Corp.) was performed in ultra-definition (UD-MS^E^)^81^ and MS^E^ mode, as established for proteome analysis of purified myelin^75,82^, to ensure correct quantification of myelin proteins that are of high abundance. Processing of LC–MS data and searching against the UniProtKB/Swiss-Prot mouse proteome database were performed using the Waters ProteinLynx Global Server v.3.0.3 with published settings^75^. For post-identification analysis including TOP3 quantification of proteins, the freely available software ISOQuant^81^ (www.isoquant.net) was used. False discovery rate for both peptides and proteins was set to a 1% threshold and only proteins reported by at least two peptides (one of which was unique) were quantified as parts per million (p.p.m.) abundance values (that is, the relative amount (w:w) of each protein in respect to the sum over all detected proteins). The Bioconductor R packages ‘limma’ and ‘*q* value’ were used to detect significant changes in protein abundance using moderated Student’s *t*-test statistics as described^83^. Optic nerve fractions from five animals per condition (10 mM glucose versus 1 mM glucose/9 mM sucrose; 10 mM glucose versus 0 mM glucose/10 mM sucrose) were processed with replicate digestion, resulting in two technical replicates per biological replicate and, thus, in a total of 20 LC–MS runs to be compared per individual experiment.

### Electron microscopy

Freshly prepared or incubated optic nerves were immersion fixed in 4% formaldehyde, 2.5% glutaraldehyde (EM-grade, Science Services) and 0.5% NaCl in phosphate buffer, pH 7.4 overnight at 4 °C. Fixed samples were embedded in EPON after dehydration with acetone as previously described^84^. Sections of 50- to 60-nm thickness were obtained with the Leica UC7 ultramicrotome equipped with a diamond knife (Histo 45° and Ultra 35 °C, Diatome) and imaged using an LEO EM 912AB electron microscope (Zeiss) equipped with an on-axis 2048 × 2048-CCD-camera (TRS).

### EM analysis

EM images from optic nerves were imported into the Fiji software. As a result of overt ultrastructural differences in incubated nerves under starvation, blinding to the conditions was not applicable here. However, analysis of GLUT1 optic nerve images was performed blinded. To create an unbiased selection of axons for which the *g*-ratio was calculated, a grid consisting of 1-μm^2^ squares for ex vivo and 4-μm^2^ squares for in vivo experiments was overlaid on each image, and axons that were crossed by the intersecting lines were selected for quantification, with the requisites of: (1) the axon being in focus and (2) the axon shape being not evidently deformed. For each axon, three circles were manually drawn around the axonal membrane, the inner layer and the outer layer of myelin. When myelin was not evenly preserved, the myelin thickness of the adjacent, preserved area was used as a proxy and the circle was corrected accordingly. The obtained area (*A*) of each circle was converted into the corresponding diameter using the formula *A* = π*r*^2^ and the *g*-ratio (outer diameter/axon diameter) was calculated. In ex vivo experiments, the obtained data from the axons with a diameter <2 μm were used for further analysis. The mean *g*-ratio for axons from one nerve was used for statistical analysis and presented as a data point in the bar graphs.

To determine the axonal size distribution in the optic nerves, axons were binned by increasing caliber and the number of counted axons for each caliber bin was divided by the total numbers of counted axons per nerve and presented as a single data point.

In optic nerves, the total number of axons and the number of degenerating and unmyelinated axons were counted in microscopic subfields of 1,445 µm^2^. Using these data, the percentage of degenerating and unmyelinated axons was calculated.

For nerves incubated in vitro, the percentage of axons containing vesicle-like structures in glial cytoplasm underneath their myelin sheath was also determined.

### Electrophysiological recording

All mice used for optic nerve electrophysiology were aged 8–12 weeks (unless otherwise stated). Recordings were performed as described previously^4,5,85^. Briefly, optic nerves were carefully dissected and quickly transferred into the recording chamber (Harvard Apparatus) and continuously superperfused with aCSF. A temperature controller (TC-10, NPI Electronic) maintained the temperature at 37 °C.

To assure the optimal stimulation and recording condition, custom-made suction electrodes were back-filled with aCSF containing 10 mM glucose. To achieve supramaximal stimulation, a battery (Stimulus Isolator 385; WPI) was used to apply a current of 0.75 mA at the proximal end of the optic nerve, to evoke a CAP at the distal end acquired by the recording electrode at 100 kHz connected to an EPC9 amplifier (Heka Elektronik). The signal was pre-amplified 10× using an Ext 10-2F amplifier (NPI Electronic) and further amplified (20–50×) and filtered at 30 kHz, using a low-noise voltage preamplifier SR560 (Stanford Research System). All recordings were done after nerve equilibration for 2 h (except for excitability and nerve conduction velocity (NCV) measurements) in aCSF containing 10 mM glucose, during which the CAP was monitored every 30 s until a stable waveform had been reached.

To measure the excitability of nerves, CAPs were evoked with currents starting from 0.05 mA and (using 0.05-mA steps) increased to 0.75 mA. The analyzed CAPA for each current was normalized to the obtained CAP at 0.75 mA. NCVs of optic nerves were determined by dividing the length of each nerve by the latency of the second peak for each nerve.

### Confocal imaging acquisition

An upright confocal laser scanning microscope (Zeiss LSM 510 META/NLO) equipped with an argon laser and a ×63 objective (Zeiss 63x IR-Achroplan, 0.9 W) was used for live imaging of the optic nerve for ATP measurement, as reported previously^5,86^. The immersion objective was placed into superfusing aCSF on top of the clamped optic nerve, with electrodes and images acquired with the time resolution of 30 s. A frame size of 114.21 ×1,33.30 μm^2^ (pinhole opening: 168 μm, pixel dwell time: 3.66 μs) was scanned (2× averaging) for cyan FP (CFP; Ex 458 nm; Em 470–500 nm), fluorescence resonance energy transfer (FRET; Ex 458 nm; Em long pass 530 nm) and YFP (Ex 514 nm; Em long pass 530 nm) channel and the focus was adjusted manually based on eye estimation of the nerve movement. To image autophagosome formation in the mouse optic nerve (aged 2–5 months), suction electrodes were used for fixing the nerve. A frame size of 133.45 × 76.19 μm^2^ was scanned for the red FP (Ex 543 nm; Em 565–615 nm) and Wasabi (Ex 488 nm; Em BP 500–550 nm) channels with the pinhole adjusted at 384 μm and the pixel dwell time: 58.4 μs.

### CAP analysis

Optic nerve function can be measured quantitatively by calculating the area underneath the evoked waveform. This CAPA represents the conduction of nearly all optic nerve axons. The evoked waveform from the optic nerve includes three peaks that represent different axons with different rates of signal speed^4,85^.

Exported CAP waveforms were analyzed for area using a customized script (available on GitHub: https://github.com/Andrea3v/CAP-waveform-analysis) in MATLAB2018b. The time between the first peak of the CAP waveform (at ∼1.2 ms after the stimulation, depending on the electrodes used) and the end of third peak (depending on the nerve length) at the last few minutes of the baseline recording was defined as the time range for CAPA integration. This window was kept constant for all recorded traces for each nerve. The calculated CAPA was then normalized to the average obtained from the last 30 min of baseline recordings. The results from several nerves were pooled, averaged and after binning plotted against time. Bar graphs depict the average CAPA for short time windows or the calculated CAPA for larger time windows. Overall, nerve conductivity was determined from the ‘CAPA area’, that is, the area under multiple CAPA curves obtained for the nerves in the experimental arm after normalizing these readings to the corresponding mean values from control nerves.

### ATP quantification

The relative level of ATP was calculated as previously reported^5^. Images were loaded in Fiji and the area of the nerve that was stable during the imaging was selected for measuring the mean intensity for three different channels: FRET, CFP and YFP. The FRET:CFP ratio was calculated to give a relative ATP concentration. The ratio was normalized to 0 and 1.0 by using the values obtained during the phase of mitochondrial blockade + glucose deprivation (5 mM azide, 0 mM glucose) and baseline (10 mM glucose), respectively. Bar graphs were obtained by averaging the values obtained at the last 5 min of each step of applied protocol for electrophysiology recordings of each nerve.

### Immunohistology

Immunostaining was performed as previously described^87^. Longitudinal cryosections of the optic nerve were fixed in 4% PFA (10–20 min) followed by washing in PBS (3× for 5 min). After 30 min of permeabilization with 0.4% Triton in PBS at room temperature (RT), blocking was performed for 30 min at RT in blocking solution (4% horse serum, 0.2% Triton in PBS). Incubation with primary antibody (Iba1 (1:1,000, cat. no. 019-19741, Wako), interleukin (IL)-33 (1:150, cat. no. AF3626, R&D Systems), Plin2 (1:150, cat. no. 15294-1-AP, Proteintech), CC1 (1:150, cat. no. OP80, Merck) and NF-L (1:150, cat. no. MCA-1D44, EnCor)) was performed in blocking solution (1% high sensitivity buffer, 0.02% Triton in PBS) at 4 °C. After washing with PBS (3× for 10 min), sections were incubated with secondary antibody in PBS/bovine serum albumin-containing DAPI (1:2,000, stock 1 mg ml^−1^) for 1 h at RT. The washed sections (3× for 10 min) in PBS were mounted and microscopy was performed.

### Autophagosome quantification

Microscopy images were processed with Fiji software and the number of autophagosomes (appearing as puncta) and cell bodies in each image were manually quantified. The total numbers of counted autophagosomes were divided by the total numbers of cell bodies in the same images. At least three images from different regions of the nerve were analyzed for each nerve and the average of the obtained values was presented as a single data point.

### Mouse behavior

All measurements were performed by the same experimenter, blinded to the animals’ genotype. Mice were trained weekly for 6 weeks before actual testing. For the Rotarod test, mice were placed on the horizontal rod. Rotation started with 1 rpm and 1 unit (rpm) was added every 10 s. The rpm value at which mice fell was recorded and the average of three repeats was reported for each mouse.

For grip strength measurements, mice were allowed to grasp a metal bar that connected to the grip strength meter. Holding on with their forelimbs, mice were slowly pulled backward until the grip was lost. The average of three measurements was reported as a data point.

### Data presentation and statistics

All data are presented as mean ± s.e.m. For cell death measurements in the optic nerve, quantifications of two to three sections from the same nerve were combined and the mean was taken as one data point (in Fig. 1c, the 16-h incubation point was determined with one to three sections). The *N* and *n* numbers indicate the total number of mice used for each condition and the total number of independently incubated/recorded optic nerves or samples that were analyzed, respectively. For proteomics after incubations at 1 mM and 10 mM glucose, two optic nerves were pooled. Statistical analysis of the data was performed in excel or Graphpad Prism 9. The experiments with big sample size were tested for data normality using D’Agostino and Pearson’s omnibus normality test and, for groups with small size, normal distribution of the data was assumed. For experiments with more than two groups ordinary one-way analysis of variance (ANOVA) or the Kruskal–Wallis test with an appropriate post-hoc test was applied for intergroup comparison. For experiments with two groups, the difference in the variance of each group of data was tested and, based on the outcome, the appropriate Student’s *t*-test (unpaired, two-tailed distribution, two-sample equal variance/homoscedastic or unequal variance/heteroscedastic) was performed. All reported *P* values in the figures are related to unpaired, homoscedastic, two-tailed Student’s *t*-test unless otherwise stated.

Animals with blindness, optic nerves with dissection artifact and unstable baseline recordings were excluded in the present study. Data collection and analysis were not performed blind to the conditions of the experiments unless otherwise stated.

### Reporting summary

Further information on research design is available in the Nature Portfolio Reporting Summary linked to this article.

## Online content

Any methods, additional references, Nature Portfolio reporting summaries, source data, extended data, supplementary information, acknowledgements, peer review information; details of author contributions and competing interests; and statements of data and code availability are available at 10.1038/s41593-024-01749-6.

## Supplementary information

Supplementary Information Supplementary Fig. 1 Raw western blots in the present study.

Reporting Summary

## Source data

Source Data Fig. 1 Plotted source data for Fig. 1. Source Data Fig. 2 Plotted source data for Fig. 2. Source Data Fig. 3 Plotted source data for Fig. 3. Source Data Fig. 4 Plotted source data for Fig. 4.

Source Data Extended Data Fig. 1 Plotted source data for Extended Data Fig. 1. Source Data Extended Data Fig. 2 Plotted source data for Extended Data Fig. 2. Source Data Extended Data Fig. 3 Plotted source data for Extended Data Fig. 3. Source Data Extended Data Fig. 4 Plotted source data for Extended Data Fig. 4. Source Data Extended Data Fig. 5 Plotted source data for Extended Data fFig. 5. Source Data Extended Data Fig. 6 Plotted source data for Extended Data Fig. 6. Source Data Extended Data Fig. 7 Plotted source data for Extended Data Fig. 7. Source Data Extended Data Fig. 8 Plotted source data for Extended Data Fig. 8.

## Data Availability

All relevant data to the manuscript will be available upon a reasonable request to corresponding authors. The MS proteomics data have been deposited to the ProteomeXchange Consortium via the PRIDE^88^ partner repository with the dataset accession no. PXD053960. Source data are provided with this paper.

## Code availability

MATLAB script for CAP analysis is available on GitHub (https://github.com/Andrea3v/CAP-waveform-analysis).
