# Author Correction: Oligodendroglial fatty acid metabolism as a central nervous system energy reserve

**DOI:** 10.1038/s41593-024-01776-3

**Published:** 2024-09-17

**Authors:** Ebrahim Asadollahi, Andrea Trevisiol, Aiman S. Saab, Zoe J. Looser, Payam Dibaj, Reyhane Ebrahimi, Kathrin Kusch, Torben Ruhwedel, Wiebke Möbius, Olaf Jahn, Jun Yup Lee, Anthony S. Don, Michelle-Amirah Khalil, Karsten Hiller, Myriam Baes, Bruno Weber, E. Dale Abel, Andrea Ballabio, Brian Popko, Celia M. Kassmann, Hannelore Ehrenreich, Johannes Hirrlinger, Klaus-Armin Nave

**Affiliations:** 1 Max Planck Institute for Multidisciplinary Sciences, Department of Neurogenetics, Göttingen, Germany; 2 University of Toronto, Sunnybrook Health Sciences Centre, Department of Physical Sciences, North York, Ontario Canada; 3 University of Zurich, Institute of Pharmacology and Toxicology, Zurich, Switzerland; 4 Center for Rare Diseases Göttingen, Department of Pediatrics and Pediatric Neurology, Georg August University Göttingen, Göttingen, Germany; 5 University of Göttingen Medical School, Institute for Auditory Neuroscience and Inner Ear Lab, Göttingen, Germany; 6 Max Planck Institute for Multidisciplinary Sciences, Department of Molecular Neurobiology, Neuroproteomics Group, Göttingen, Germany; 7 University Medical Center Göttingen, Department of Psychiatry and Psychotherapy, Translational Neuroproteomics Group, Göttingen, Germany; 8 School of Medical Sciences and Charles Perkins Centre, The University of Sydney, Camperdown, New South Wales Australia; 9 Department for Bioinformatics and Biochemistry, Braunschweig Integrated Center of System Biology, Technische Universität Braunschweig, Braunschweig, Germany; 10 Lab of Cell Metabolism, Department of Pharmaceutical and Pharmacological Sciences, KU Leuven, Leuven, Belgium; 11 Department of Medicine, David Geffen School of Medicine at UCLA, Los Angeles, CA USA; 12 Telethon Institute of Genetics and Medicine, Naples, Italy; 13 Department of Translational Medical Sciences, Federico II University, Naples, Italy; 14 Department of Molecular and Human Genetics, Baylor College of Medicine, Houston, TX USA; 15 Jan and Dan Duncan Neurological Research Institute, Texas Children’s Hospital, Houston, TX USA; 16 Feinberg School of Medicine, Northwestern University, Chicago, IL USA; 17 Max Planck Institute for Multidisciplinary Sciences, Clinical Neuroscience, Göttingen, Germany; 18 Central Institute of Mental Health, Mannheim, Germany; 19 Carl-Ludwig-Institute for Physiology, Faculty of Medicine, University of Leipzig, Leipzig, Germany

**Keywords:** Oligodendrocyte, Cellular neuroscience

Correction to: *Nature Neuroscience* 10.1038/s41593-024-01749-6, published online 9 September 2024.

In the version of the article initially published, Andrea Ballabio’s surname appeared incorrectly (as Balabio) and has now been amended in the HTML and PDF versions of the article.

