# Supplementary material for: Oligodendroglial fatty acid metabolism as a central nervous system energy reserve
Source: Nat Neurosci. 2024 Sep 9;27(10):1934–44. doi: 10.1038/s41593-024-01749-6 (PMC11452346; doi:10.1038/s41593-024-01749-6)

nature neuroscience

Supplementary information

https://doi.org/10.1038/s41593-024-01749-6

Oligodendroglial fatty acid metabolism as a central nervous system energy reserve

In the format provided by the authors and unedited

Fig.4 b

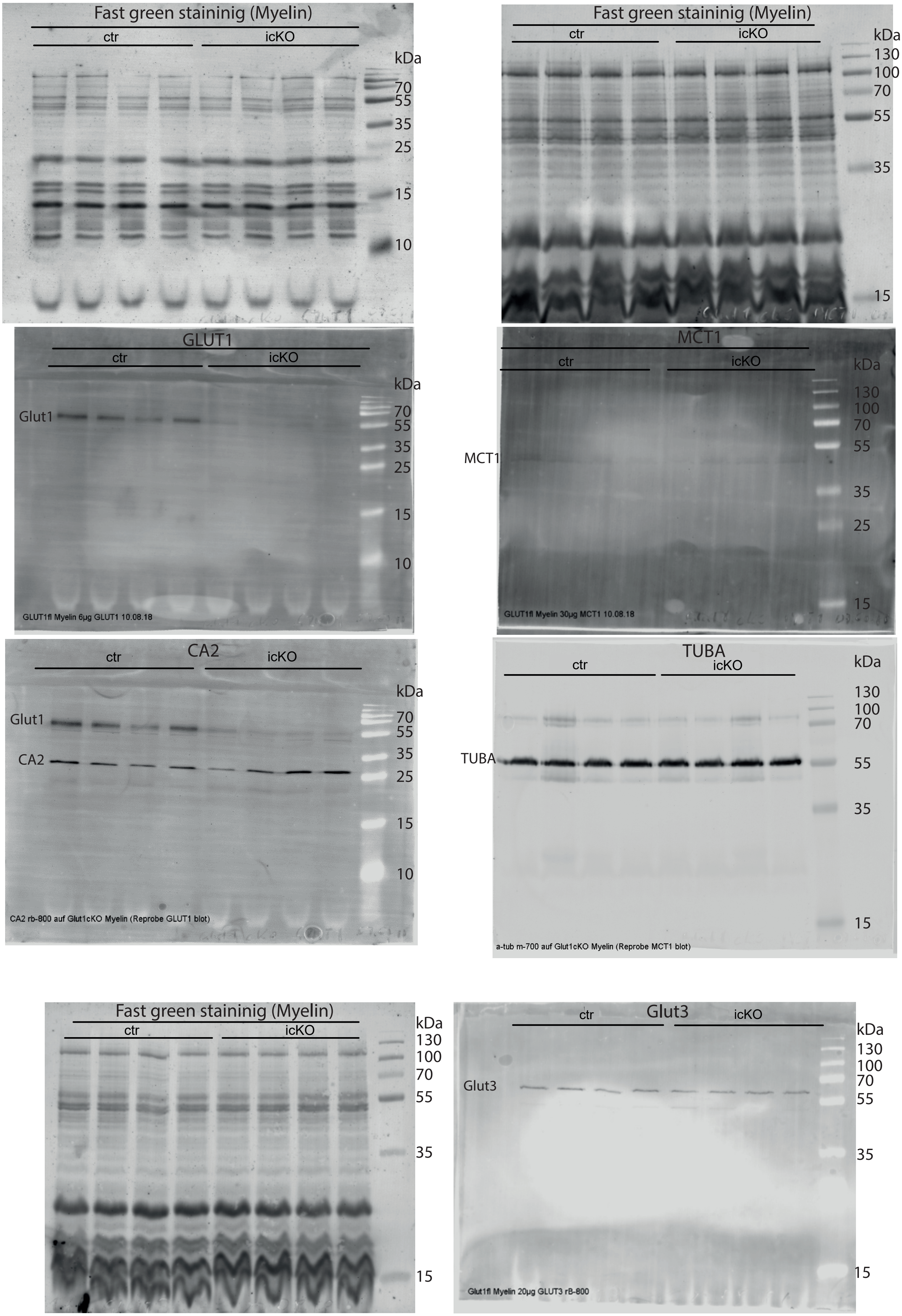
Fast green staininig (Myelin)
ctr
icKO
kDa
70
55
35
25
15
10
Fast green staininig (Myelin)
ctr
icKO
kDa
130
100
70
55
35
15
GLUT1
ctr
icKO
kDa
Glut1
70
55
35
25
15
10
GLUT1fl Myelin 6µg GLUT1 10.08.18
MCT1
ctr
icKO
kDa
130
100
70
55
MCT1
35
25
15
GLUT1fl Myelin 30µg MCT1 10.08.18
CA2
ctr
icKO
kDa
Glut1
70
55
CA2
35
25
15
10
CA2 rb-800 auf Glut1cKO Myelin (Reprobe GLUT1 blot)
TUBA
ctr
icKO
kDa
130
100
70
TUBA
55
35
15
a-tub m-700 auf Glut1cKO Myelin (Reprobe MCT1 blot)
Fast green staininig (Myelin)
ctr
icKO
kDa
130
100
70
55
35
15
Glut3
ctr
icKO
kDa
130
100
70
Glut3
55
35
15
Glut1fl Myelin 20µg GLUT3 rB-800

Fig.4 i

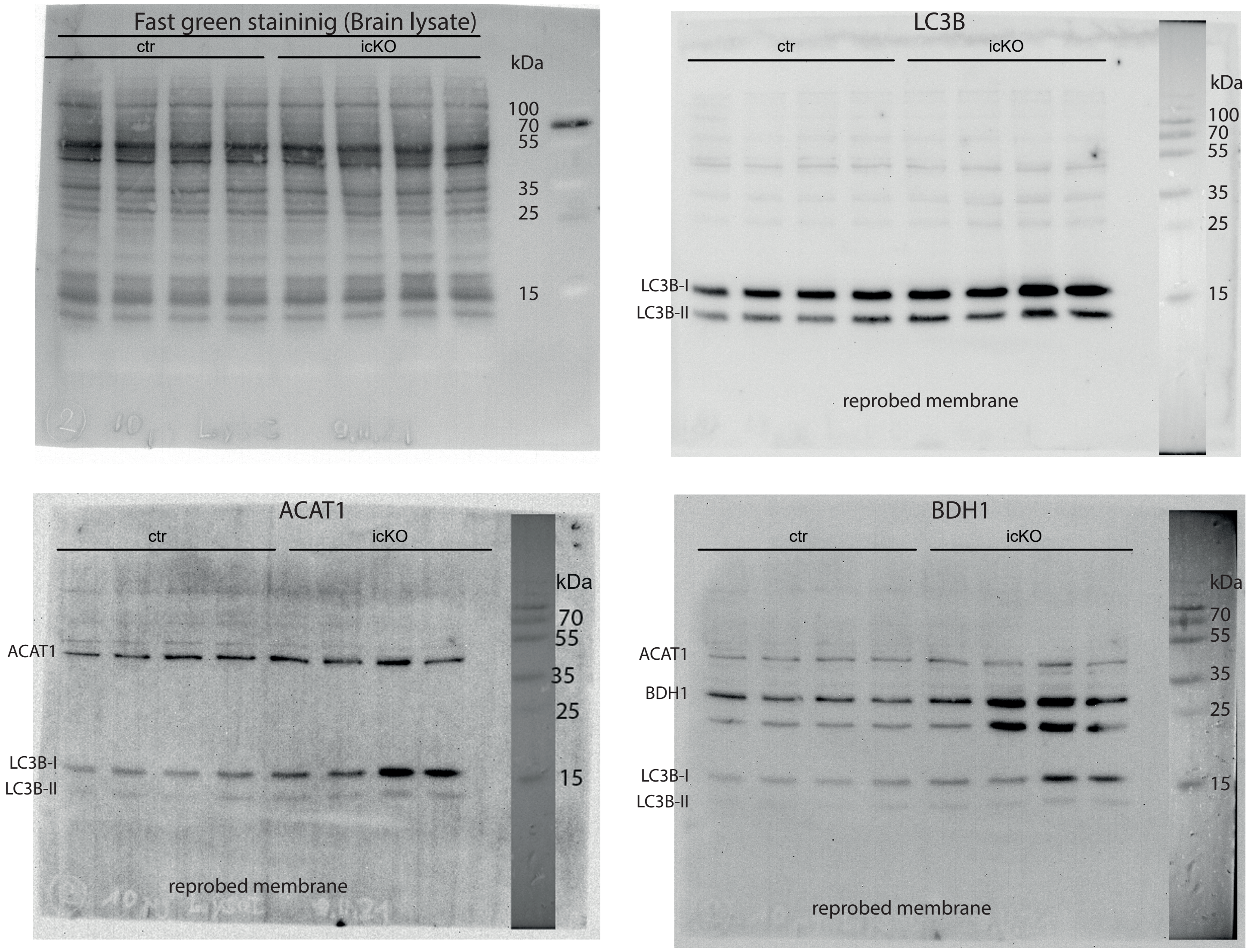

Fast green staininig (Brain lysate)
ctr
icKO
kDa
100
70
55
35
25
15
LC3B
ctr
icKO
kDa
100
70
55
35
25
15
LC3B-I
LC3B-II
reprobed membrane
ACAT1
ctr
icKO
kDa
70
55
35
25
15
ACAT1
LC3B-I
LC3B-II
reprobed membrane
BDH1
ctr
icKO
kDa
70
55
35
25
15
ACAT1
BDH1
LC3B-I
LC3B-II
reprobed membrane

Extended Data fig.3 c

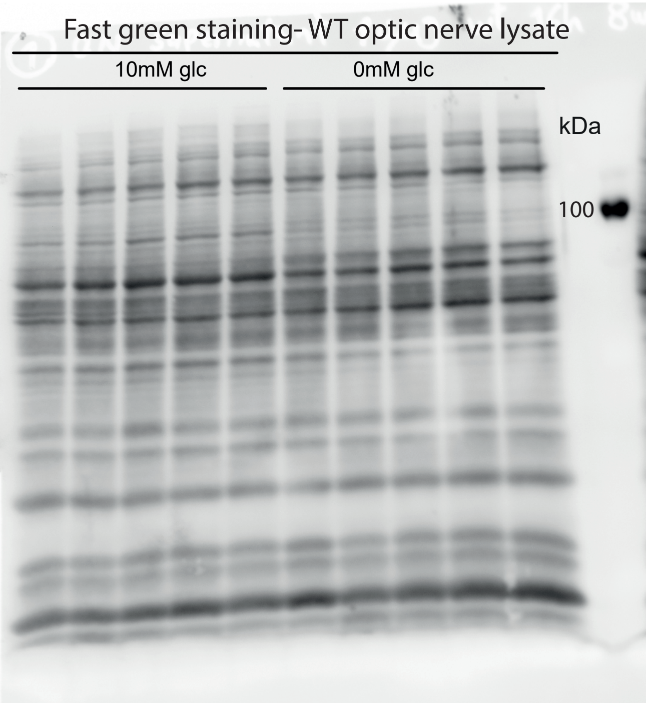

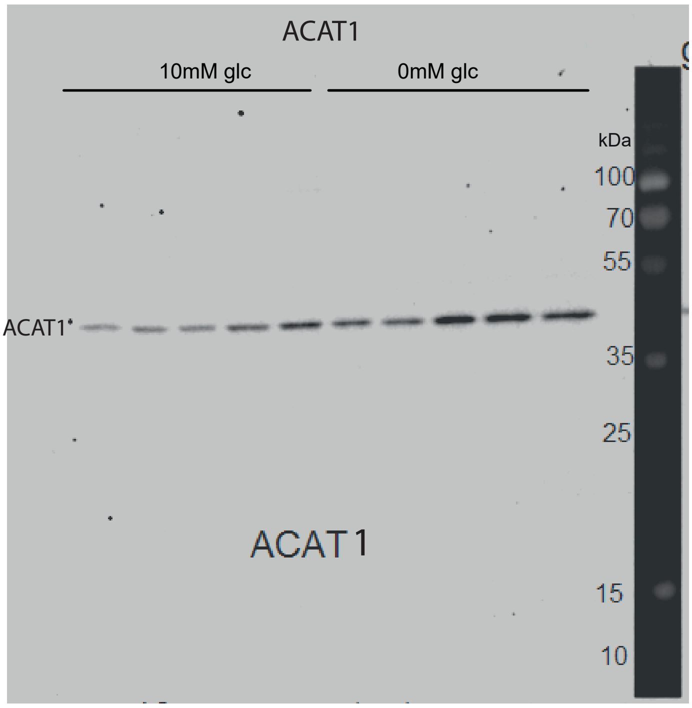

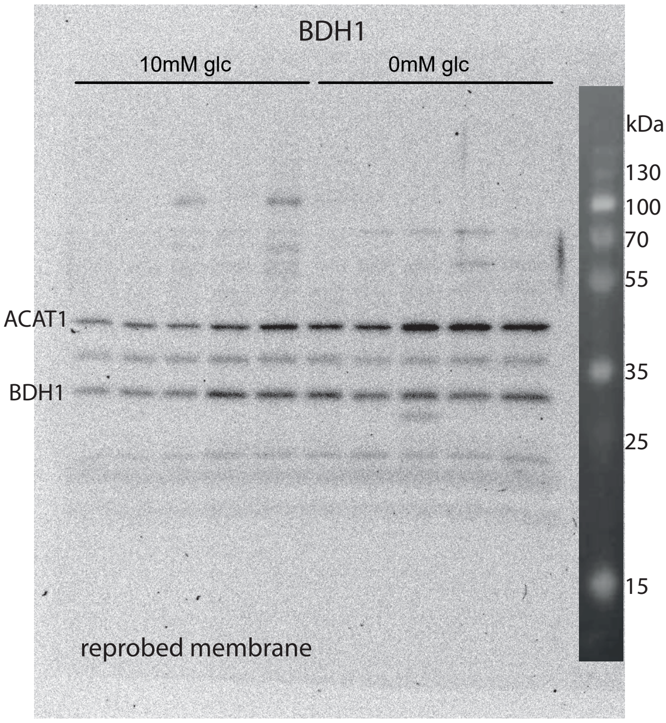

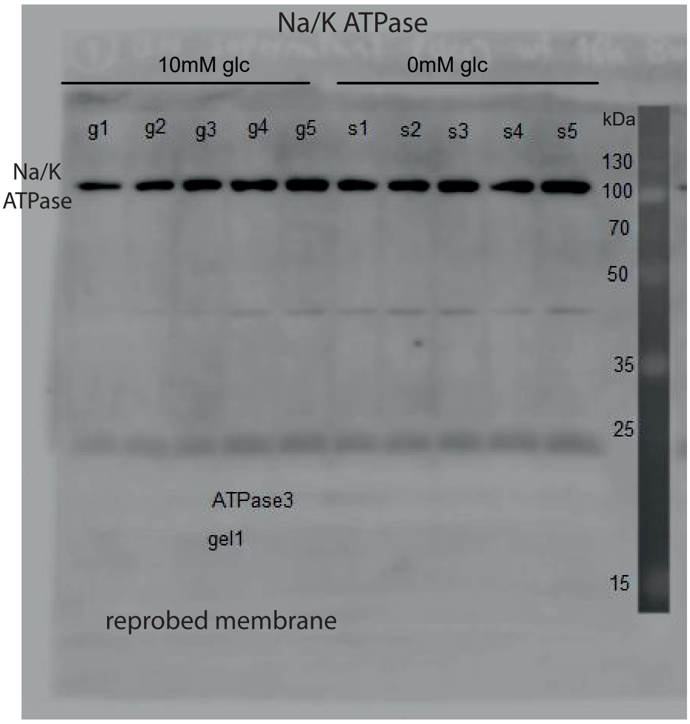

Supplement: Supplementary file 1 — Supplementary Fig. 1 Raw western blots in the present study. [file 41593_2024_1749_MOESM1_ESM.pdf]
