# Supplementary material for: Oligodendroglial fatty acid metabolism as a central nervous system energy reserve
Source: Nat Neurosci. 2024 Sep 9;27(10):1934–44. doi: 10.1038/s41593-024-01749-6 (PMC11452346; doi:10.1038/s41593-024-01749-6)
Supplement: Supplementary file 2 — Reporting Summary [file 41593_2024_1749_MOESM2_ESM.pdf]

nature portfolio

Corresponding author(s): Klaus-Armin Nave
Ebrahim Asadollahi

Last updated by author(s): Jul 10, 2024

Reporting Summary

Nature Portfolio wishes to improve the reproducibility of the work that we publish. This form provides structure for consistency and transparency in reporting. For further information on Nature Portfolio policies, see our Editorial Policies and the Editorial Policy Checklist.

Statistics

For all statistical analyses, confirm that the following items are present in the figure legend, table legend, main text, or Methods section.

n/a	Confirmed	
☐	☒	The exact sample size (n) for each experimental group/condition, given as a discrete number and unit of measurement
☐	☒	A statement on whether measurements were taken from distinct samples or whether the same sample was measured repeatedly
☐	☒	The statistical test(s) used AND whether they are one- or two-sided<br>*Only common tests should be described solely by name; describe more complex techniques in the Methods section.*
☒	☐	A description of all covariates tested
☐	☒	A description of any assumptions or corrections, such as tests of normality and adjustment for multiple comparisons
☐	☒	A full description of the statistical parameters including central tendency (e.g. means) or other basic estimates (e.g. regression coefficient) AND variation (e.g. standard deviation) or associated estimates of uncertainty (e.g. confidence intervals)
☐	☒	For null hypothesis testing, the test statistic (e.g. F, t, r) with confidence intervals, effect sizes, degrees of freedom and P value noted<br>*Give P values as exact values whenever suitable.*
☒	☐	For Bayesian analysis, information on the choice of priors and Markov chain Monte Carlo settings
☒	☐	For hierarchical and complex designs, identification of the appropriate level for tests and full reporting of outcomes
☐	☒	Estimates of effect sizes (e.g. Cohen's d, Pearson's r), indicating how they were calculated

Our web collection on statistics for biologists contains articles on many of the points above.

Software and code

Policy information about availability of computer code

Data collection	Ex vivo electrophysiology: CAP waveforms were acquired using Patchmaster software (v2.15 or V2x90.3).<br>Epifluorescence: Images were acquired using ZEN2 software (Blue edition, version:2.0.0.0, Zeiss, Germany).<br>Confocal microscopy(Zeiss 510 Meta NLO): ZEN 2009.<br>Western blot: imaging was performed using a near-infrared fluorescence scanner (Odyssey, Li-cor, image studio version 3.1.4).<br>Electron microscopy: EM images were acquired iTEM software 5.2(Build 6075, Olympus soft imaging solutions, Münster, Germany).<br>Proteomics: Waters MassLynx 4.1 was used.
Data analysis	Electrophysiology: exported CAP waveforms were analyzed for area using a custom-script in MATLAB2018b.<br>Cell death quantification: tif files were opened in imagej (version 2.0.0) and using a plugin further analysis for colocalization was performed in Imaris (8.1.2)<br>ATP measurements: the LSM files were imported to Imagej(version 2.0.0) for intensity measurement and obtained data were analysed further using excel(Microsoft office).<br>G-ratio: the tif files were analyzed in Imagej and obtained data were analysed further in Excel (Microsoft office).<br>quantification of protein abundance: images obtained from the near-infrared fluorescence imager were analyzed using the Image Studio software (Image studio version 3.1.4).<br>Proteomics: Waters ProteinLynx Global Server 3.0, IsoQuant (www.isoquant.net), Bioconductor R-packages limma and q-value<br>Statistical analysis: Graphpad Prism 9

For manuscripts utilizing custom algorithms or software that are central to the research but not yet described in published literature, software must be made available to editors and reviewers. We strongly encourage code deposition in a community repository (e.g. GitHub). See the Nature Portfolio guidelines for submitting code & software for further information.

Data

Policy information about availability of data

All manuscripts must include a data availability statement. This statement should provide the following information, where applicable:

- Accession codes, unique identifiers, or web links for publicly available datasets
- A description of any restrictions on data availability
- For clinical datasets or third party data, please ensure that the statement adheres to our policy

The data used to make the figures will be made available upon request to the corresponding authors. All proteomics data is deposited in PRIDE.

Research involving human participants, their data, or biological material

Policy information about studies with human participants or human data. See also policy information about sex, gender (identity/presentation), and sexual orientation and race, ethnicity and racism.

Reporting on sex and gender	N/A
Reporting on race, ethnicity, or other socially relevant groupings	N/A
Population characteristics	N/A
Recruitment	N/A
Ethics oversight	N/A

Note that full information on the approval of the study protocol must also be provided in the manuscript.

Field-specific reporting

Please select the one below that is the best fit for your research. If you are not sure, read the appropriate sections before making your selection.

☒ Life sciences ☐ Behavioural & social sciences ☐ Ecological, evolutionary & environmental sciences

For a reference copy of the document with all sections, see nature.com/documents/nr-reporting-summary-flat.pdf

Life sciences study design

All studies must disclose on these points even when the disclosure is negative.

Sample size

We did not perform sample-size calculation.However, we used the standard sample sizes used currently in the literature for electrophysiology (Trevisiol, A., et al. Monitoring ATP dynamics in electrically active white matter tracts. Elife 6, e24241 (2017)) and g-ratio analysis (Sun, L.O., et al. Spatiotemporal control of CNS myelination by oligodendrocyte programmed cell death through the TFEB-PUMA axis. Cell 175, 1811-1826. e1821 (2018)).

Data exclusions

Regarding the cell death measurements, the nerves that had not been dissected properly were excluded from quantifications.
For electrophysiological recording, all the data with stable baseline were included in our calculations. However, in rare cases baseline was not stable and the CAP of the optic nerve starts to increase during recording that is due to changes in resistance of electrodes (technical problem and not biologically relevant) the recordings were excluded from our calculations.
In ATP measurements, since live imaging of the optic nerve is performed in a chamber with constant flow of aCSF, small nerve movements could greatly affect the imaging. Therefore, acquired images from more stable nerves were included in the calculations.

Replication

All the data related to optic nerve incubation were obtained from independent experiments as indicated in figur's legend with n.
Proteomics data obtained from independent experiments at two different starvation conditions (First experiment: 1mM vs 10mM glucose; second experiment: 0mM vs 10mM glucose) and expression changes in starved nerves in comparison to expression level in related 10mM glucose were calculated.
Electrophysiology recording under starvation condition in the presence of peroxisomal and mitochondrial inhibitors (4-Br and Thio) and MFP2 cKO mice had been repeated using another protocol and similar differences in CAP were observed.
Regarding the optic nerve incubation from PCNP-RFP-Wasabi-LC3 mouse line, in independent experiments and transient formation of autophagosomes was observed at around 8:30h incubation (the scan rate was different among the presented images in the paper to acquire higher quality images).

Randomization

All the wild type animals ordered from the animal facility and we did not have any involvement in choosing the mice except for their gender that we used both male and females. Regarding the transgenic or reporter lines, most of the involved control animals were littermate controls and allocation of animals was based on the littermate genotypes.

Blinding

For quantifying the dead cells in incubated optic nerves the blinded quantification was not applicable due to dramatic differences between starved nerves vs controls.
For g-ratio measurements of incubated optic nerves ex vivo blinded measurements was not possible because of differences in nerve preservation during incubation.
For ex vivo electrophysiology data, data acquisition was done automatically using the patchmaster software and only switching between different solutions were done manually and the area under the three peaks were always calculated and were normalized to last minuets of baseline. Therefore, blinding does not apply here.
G-ratio measurements for optic nerves of GLUT1 knock out mice was performed blinded.
We were blinded to histological analysis of GLUT1 immunostained sections.
EM and histological analysis of MFP2 conditional KO mice were performed blinded.

Reporting for specific materials, systems and methods

We require information from authors about some types of materials, experimental systems and methods used in many studies. Here, indicate whether each material, system or method listed is relevant to your study. If you are not sure if a list item applies to your research, read the appropriate section before selecting a response.

Materials & experimental systems

n/a	Involved in the study
☐	☒ Antibodies
☒	☐ Eukaryotic cell lines
☒	☐ Palaeontology and archaeology
☐	☒ Animals and other organisms
☒	☐ Clinical data
☒	☐ Dual use research of concern
☒	☐ Plants

Methods

n/a	Involved in the study
☒	☐ ChIP-seq
☒	☐ Flow cytometry
☒	☐ MRI-based neuroimaging

Antibodies

Antibodies used

CC1 (APC) (1:150, OP80, Merck)
Iba1 (1:1000, 019-19741, Wako)
Plin2 (1:150, 15294-1-AP, Proteintech)
ACAT1 (1:3000, 16215-1-AP, proteintech),
IL-33 (1:150, AF3626, R&D systems)
BDH1 (1:500, 15417-1-AP, proteintech),
LC3B (1:2000, NB100-2220, Novusbio),
HRP conjugated secondary antibodies (1:5000, 115-03-003 and 111-035-003, Dianova),
Na+/K+ATPaseα1 (1:1000, ab7671, Abcam),
GLUT1 (1:1000, Berghoff et al., 2017),
GLUT3 (1:1000, ab191071, abcam),
MCT1 (1:1000, Stumpf et al., 2019),
carbonic anhydrase 2 (CA2, 1:1000, Ghandour et al. 1980)
α-tubulin (TUBA, 1:1000, T 5168, Sigma)
NF-L: (1:150, MCA-1D44, EnCor)
Mouse IgG (H&L) Antibody Dylight™ 680 Conjugated, 610-144-002; Rockland (1:10000)
Rabbit IgG (H&L) Antibody DyLight™ 800 Conjugated, 611-145-002; Rockland (1:10000)

Validation

Antibodies except for GLUT1 and MCT1, used in this study are commercially available and have been validated by the manufacturers (see technical data sheets accessible on the manufacturers' websites).Validation for GLUT1 and MCT1 antibodies was done by peptide competition and for MCT1 using the MCT1 het mice.

References:

GLUT1: Berghoff SA, Düking T, Spieth L, Winchenbach J, Stumpf SK, Gerndt N, Kusch K, Ruhwedel T, Möbius W, Saher G. Blood-brain barrier hyperpermeability precedes demyelination in the cuprizone model. Acta Neuropathol Commun. 2017 Dec 1;5(1):94. doi: 10.1186/s40478-017-0497-6. PMID: 29195512; PMCID: PMC5710130.

MCT1: Stumpf SK, Berghoff SA, Trevisiol A, Spieth L, Düking T, Schneider LV, Schlaphoff L, Dreha-Kulaczewski S, Bley A, Burfeind D, Kusch K, Mitkovski M, Ruhwedel T, Guder P, Röhse H, Denecke J, Gärtner J, Möbius W, Nave KA, Saher G. Ketogenic diet ameliorates axonal defects and promotes myelination in Pelizaeus-Merzbacher disease. Acta Neuropathol. 2019 Oct;138(4):673-674. doi: 10.1007/s00401-019-02064-2. Epub 2019 Sep 3. PMID: 31482207; PMCID: PMC6778063.

CA2: Ghandour MS, Langley OK, Vincendon G, Gombos G, Filippi D, Limozin N, Dalmasso D, Laurent G. Immunochemical and immunohistochemical study of carbonic anhydrase II in adult rat cerebellum: a marker for oligodendrocytes. Neuroscience. 1980;5(3):559-71. doi: 10.1016/0306-4522(80)90053-6. PMID: 6769072.

GLUT3: Jawale CV, Ramani K, Li DD, Coleman BM, Oberoi RS, Kupul S, Lin L, Desai JV, Delgoffe GM, Lionakis MS, Bender FH, Prokopienko AJ, Nolin TD, Gaffen SL, Biswas PS. Restoring glucose uptake rescues neutrophil dysfunction and protects against

systemic fungal infection in mouse models of kidney disease. Sci Transl Med. 2020 Jun 17;12(548):eaay5691. doi: 10.1126/scitranslmed.aay5691. PMID: 32554707; PMCID: PMC7879380.

α-tubulin: Hossain MM, Richardson JR. Nerve Growth Factor Protects Against Pyrethroid-Induced Endoplasmic Reticulum (ER) Stress in Primary Hippocampal Neurons. Toxicol Sci. 2020 Mar 1;174(1):147-158. doi: 10.1093/toxsci/kfz239. PMID: 31841155; PMCID: PMC7043229.

LC3B: Tran S, Juliani J, Harris TJ, Evangelista M, Ratcliffe J, Ellis SL, Baloyan D, Reehorst CM, Nightingale R, Luk IY, Jenkins LJ, Ghilas S, Yakou MH, Inguanti C, Johnson C, Buchert M, Lee JC, De Cruz P, Duszyc K, Gleeson PA, Kile BT, Mielke LA, Yap AS, Mariadason JM, Douglas Fairlie W, Lee EF. BECLIN1 is essential for intestinal homeostasis involving autophagy-independent mechanisms through its function in endocytic trafficking. Commun Biol. 2024 Feb 20;7(1):209. doi: 10.1038/s42003-024-05890-7. PMID: 38378743; PMCID: PMC10879175.

ACAT1: Zhang L, Zhang Z, Li C, Zhu T, Gao J, Zhou H, Zheng Y, Chang Q, Wang M, Wu J, Ran L, Wu Y, Miao H, Zou X, Liang B. S100A11 Promotes Liver Steatosis via FOXO1-Mediated Autophagy and Lipogenesis. Cell Mol Gastroenterol Hepatol. 2021;11(3):697-724. doi: 10.1016/j.jcmgh.2020.10.006. Epub 2020 Oct 17. PMID: 33075563; PMCID: PMC7841444.

BDH1: Düking T, Spieth L, Berghoff SA, Piepkorn L, Schmidke AM, Mitkovski M, Kannaiyan N, Hosang L, Scholz P, Shaib AH, Schneider LV, Hesse D, Ruhwedel T, Sun T, Linhoff L, Trevisiol A, Köhler S, Pastor AM, Misgeld T, Sereda M, Hassouna I, Rossner MJ, Odoardi F, Ischebeck T, de Hoz L, Hirrlinger J, Jahn O, Saher G. Ketogenic diet uncovers differential metabolic plasticity of brain cells. Sci Adv. 2022 Sep 16;8(37):eabo7639. doi: 10.1126/sciadv.abo7639. Epub 2022 Sep 16. PMID: 36112685; PMCID: PMC9481126.

Na+/K ATPase: Edmunds LR, Huckestein BR, Kahn M, Zhang D, Chu Y, Zhang Y, Wendell SG, Shulman GI, Jurczak MJ. Hepatic insulin sensitivity is improved in high-fat diet-fed Park2 knockout mice in association with increased hepatic AMPK activation and reduced steatosis. Physiol Rep. 2019 Nov;7(21):e14281. doi: 10.14814/phy2.14281. PMID: 31724300; PMCID: PMC6854109.

Iba1: Thumu SCR, Jain M, Soman S, Das S, Verma V, Nandi A, Gutmann DH, Jayaprakash B, Nair D, Clement JP, Marathe S, Ramanan N. SRF-deficient astrocytes provide neuroprotection in mouse models of excitotoxicity and neurodegeneration. Elife. 2024 Feb 9;13:e95577. doi: 10.7554/eLife.95577. Erratum in: Elife. 2024 Jul 01;13:e101107. doi: 10.7554/eLife.101107. PMID: 38289036; PMCID: PMC10857791.

Plin2: Cui D, Wang Z, Dang Q, Wang J, Qin J, Song J, Zhai X, Zhou Y, Zhao L, Lu G, Liu H, Liu G, Liu R, Shao C, Zhang X, Liu Z. Spliceosome component Usp39 contributes to hepatic lipid homeostasis through the regulation of autophagy. Nat Commun. 2023 Nov 3;14(1):7032. doi: 10.1038/s41467-023-42461-6. PMID: 37923718; PMCID: PMC10624899.

CC1: Bhat RV, Axt KJ, Fosnaugh JS, Smith KJ, Johnson KA, Hill DE, Kinzler KW, Baraban JM. Expression of the APC tumor suppressor protein in oligodendroglia. Glia. 1996 Jun;17(2):169-74. doi: 10.1002/(SICI)1098-1136(199606)17:2<169::AID-GLIA8>3.0.CO;2-Y. PMID: 8776583.

IL-33: Wei H, Zhen L, Wang S, Yang L, Zhang S, Zhang Y, Jia P, Wang T, Wang K, Zhang Y, Ma L, Lv J, Zhang P. Glyceryl triacetate promotes blood-brain barrier recovery after ischemic stroke through lipogenesis-mediated IL-33 in mice. J Neuroinflammation. 2023 Nov 15;20(1):264. doi: 10.1186/s12974-023-02942-3. PMID: 37968698; PMCID: PMC10648711.

NF-L: Bacioglu M, Maia LF, Preische O, Schelle J, Apel A, Kaeser SA, Schweighauser M, Eninger T, Lambert M, Pilotto A, Shimshek DR, Neumann U, Kahle PJ, Staufenbiel M, Neumann M, Maetzler W, Kuhle J, Jucker M. Neurofilament Light Chain in Blood and CSF as Marker of Disease Progression in Mouse Models and in Neurodegenerative Diseases. Neuron. 2016 Jul 6;91(1):56-66. doi: 10.1016/j.neuron.2016.05.018. Epub 2016 Jun 9. Erratum in: Neuron. 2016 Jul 20;91(2):494-496. doi: 10.1016/j.neuron.2016.07.007. PMID: 27292537.

Animals and other research organisms

Policy information about studies involving animals; ARRIVE guidelines recommended for reporting animal research, and Sex and Gender in Research

Laboratory animals	All the used mice in this study were on a C57BL/6 background except the ALDG (reporter) mouse line that has a mixed background obtained from breeding C57BL/6 with FVB/N mice.<br>The animals used in this study were male or female at 8-10 weeks old, except for glut1 icKO mice that were used at seven months of age and the pCNP-RFP-Wasabi- LC3 mouse line that were used at the age of 8-24 weeks. Mice were group-housed in the local animal facility of the Max Planck Institute for Multidisciplinary Sciences under a 12-h dark/12-h light cycle and fed ad-libitum (temperature of 22°C, 30–70% humidity).
Wild animals	This study did not involve wild animals.
Reporting on sex	Both male and female animals were used in this study. Acquired data for each experimental condition showing great overlap between different genders. Therefore, we did not do further statistical analysis.
Field-collected samples	This study did not involve Field-collected samples.
Ethics oversight	The experimental procedure were approved and performed in accordance with The Niedersächsisches Landesamt für Verbraucherschutz und Lebensmittelsicherheit (LAVES); License number 18/2962

Note that full information on the approval of the study protocol must also be provided in the manuscript.
